# Estimation of kinship coefficient in structured and admixed populations using sparse sequencing data

**DOI:** 10.1371/journal.pgen.1007021

**Published:** 2017-09-29

**Authors:** Jinzhuang Dou, Baoluo Sun, Xueling Sim, Jason D. Hughes, Dermot F. Reilly, E. Shyong Tai, Jianjun Liu, Chaolong Wang

**Affiliations:** 1 Computational and Systems Biology, Genome Institute of Singapore, Singapore, Singapore; 2 Saw Swee Hock School of Public Health, National University of Singapore, Singapore, Singapore; 3 Genetics, Merck Sharp & Dohme Corp., Kenilworth, New Jersey, United States of America; 4 Duke-NUS Medical School, National University of Singapore, Singapore, Singapore; 5 Yong Loo Lin School of Medicine, National University of Singapore, Singapore, Singapore; 6 Human Genetics, Genome Institute of Singapore, Singapore, Singapore; University of Washington, UNITED STATES

## Abstract

Knowledge of biological relatedness between samples is important for many genetic studies. In large-scale human genetic association studies, the estimated kinship is used to remove cryptic relatedness, control for family structure, and estimate trait heritability. However, estimation of kinship is challenging for sparse sequencing data, such as those from off-target regions in target sequencing studies, where genotypes are largely uncertain or missing. Existing methods often assume accurate genotypes at a large number of markers across the genome. We show that these methods, without accounting for the genotype uncertainty in sparse sequencing data, can yield a strong downward bias in kinship estimation. We develop a computationally efficient method called SEEKIN to estimate kinship for both homogeneous samples and heterogeneous samples with population structure and admixture. Our method models genotype uncertainty and leverages linkage disequilibrium through imputation. We test SEEKIN on a whole exome sequencing dataset (WES) of Singapore Chinese and Malays, which involves substantial population structure and admixture. We show that SEEKIN can accurately estimate kinship coefficient and classify genetic relatedness using off-target sequencing data down sampled to ~0.15X depth. In application to the full WES dataset without down sampling, SEEKIN also outperforms existing methods by properly analyzing shallow off-target data (~0.75X). Using both simulated and real phenotypes, we further illustrate how our method improves estimation of trait heritability for WES studies.

## Author summary

Inference of genetic relatedness from molecular markers has broad applications in many areas, including quantitative genetics, forensics, evolution and ecology. Classic estimators, however, are not suitable for low-coverage sequencing data, which have high levels of genotype uncertainty and missing data. We evaluate existing methods and describe a new method for kinship estimation using sparse sequencing data. Our method leverages correlations between neighboring markers and models genotype uncertainty in kinship estimators for both homogeneous populations and admixed populations. We show that our method can accurately estimate kinship coefficient even when the sequencing depth is as low as ~0.15X, while existing methods have strong downward bias. Our method can be applied to estimate kinship using sparse off-target data and thus enables control of family structure and estimation of heritability in target sequencing studies, in which the deeply sequenced target regions are often too small to infer genetic relatedness. Even for whole exome sequencing, we show that our method can improve kinship and heritability estimation by including off-target data, compared to conventional analyses solely based on the target regions.

## Introduction

Understanding biological relatedness plays a central role in quantitative genetic studies of heritable traits and diseases. For example, complete pedigree information is required for linkage analysis and family-based association studies. In population-based association studies, inference of genetic relatedness is a routine practice in quality control because cryptic relatedness is a major confounding factor that can lead to spurious association signals. The estimated pairwise relatedness matrix is often used to model phenotype covariance through mixed models for both quantitative traits [1–3] and case-control studies [4]. Such mixed model approaches have been widely used to control for population and family structure in association tests [1–4] and to estimate heritability for traits of interests [5,6]. Genetic relatedness between samples can also be leveraged to improve imputation of missing phenotypes and thus boost the statistical power of multiple-phenotype association studies [7,8]. In addition to quantitative genetics, inference of genetic relatedness has broad applications in many other areas, including forensics, agriculture, evolution, and ecology [9].

Kinship coefficient, defined as the probability that two homologous alleles drawn from each of two individuals are identical by descent (IBD), is a classic measurement of relatedness [10,11]. While kinship coefficients can be derived from pedigree, many estimators based on the maximum likelihood method or the method of moments have been developed to estimate kinship coefficients from genotype data, especially in population-based studies in which pedigree information is not available or inaccurate. While likelihood estimators [12–14] are powerful to test the hypothesized relationships, moment estimators [15–17] are widely used due to their computational efficiencies in large datasets. Two popular moment estimators that assume random mating in a homogeneous sample have been implemented in the KING [18] and GCTA [5] programs. These homogeneous estimators, however, can produce biased estimation in the presence of population structure [13,18,19]. Such bias might be corrected by modeling the drift of allele frequencies in the subpopulation where both individuals come from [13,19]. While KING has a robust estimator (KING-rob) for samples with population structure, it does not perform well in analyzing admixed samples, in which two related individuals might have different ancestry background [20,21]. Two moment estimators, REAP [20] and PC-Relate [21], and a likelihood estimator, RelateAdmix [22], have been proposed for kinship estimation in admixed samples. These methods account for different ancestry background of admixed individuals using individual-specific allele frequencies derived from either model-based methods for population structure analysis, such as ADMIXTURE [23,24], or principal components analysis (PCA) [25].

These existing kinship estimators require accurate genotype data across genome-wide SNPs, which may not be available in next-generation sequencing studies. The shallow whole-genome sequencing design is widely used in large population-based studies, in which individual genotypes might be inaccurate but the statistical power for association tests is optimized as the sample size increases [26–28]. Additionally, due to sample quality, shallow sequencing data are typical from studies of wild animals, forensics, and ancient human DNA [29–31]. Target sequencing is another widely used design in human genetic studies by focusing on candidate loci of interests or the whole exome [32–37]. More than 60,000 exomes from over 20 studies have been contributed to the Exome Aggregation Consortium (ExAC) Browser [36]. In target sequencing studies, accurate genotypes are only available for the deeply-sequenced target regions, which often do not have enough SNPs to infer either individual ancestry or pairwise genetic relatedness, posting a limitation to control for major confounding factors of population structure and family relatedness. The vast off-target regions are typically covered by ~0.1-1X sequence reads, which are byproducts of target sequencing due to imperfect capture technologies. We have developed a method called LASER that can utilize the off-target reads to accurately infer an individual’s genetic ancestry background [38,39]. Estimation of pairwise relatedness remains challenging because the analysis requires both individuals to have data across a common set of SNPs, which are very few because off-target reads are sparse. For example, if each individual have ~10% of their off-target SNPs covered by some reads, there will be only ~1% (= 0.1^2^) of SNPs sequenced in both individuals. Furthermore, there is huge genotype uncertainty at these SNPs due to extremely low sequencing depth. Recently, a likelihood method called lcMLkin has been proposed to estimate kinship from shallow sequencing data by explicitly modeling the uncertainty [40]. However, lcMLkin assumes Hardy-Weinberg equilibrium (HWE) and thus cannot be applied to samples with population structure and admixture.

In this paper, we develop a new method called SEEKIN (SEquence-based Estimation of KINship) to estimate kinship using sparse sequence reads. The key rationale is that even though the number of SNPs sequenced in both of a pair of individuals is small, neighboring SNPs in the genome are often correlated due to linkage disequilibrium (LD). With large amounts of existing whole genome sequencing (WGS) data, such as the 1000 Genomes Project [28], we can leverage LD to call genotypes with probabilities across majority of the SNPs in each individual, including SNPs that are not even sequenced [41]. Such an approach has been implemented in many phasing and imputation programs, which are widely used in genome-wide association studies (GWAS) [42–45]. Through imputation, we can substantially increase the number of SNPs shared by any two individuals, thereby making it possible to estimate pairwise relatedness. We model the genotype uncertainty [46] and propose two moment estimators of kinship; one for homogeneous samples and the other for heterogeneous samples with population structure and admixture. We evaluate our method using whole-exome sequencing (WES) and array genotyping data for 762 related individuals from the Singapore Living Biobank Project, which include Chinese and Malays with substantial amount of admixture. We show that our method can accurately estimate kinship coefficient for both homogeneous and heterogeneous samples even when the sequencing depth is as low as ~0.15X, while existing methods show strong downward bias. Compared to results based on high-coverage target regions in WES, which are ~1.5% of the genome, our method also improves kinship estimation and the subsequent heritability estimation by properly utilizing data from off-target regions. While SEEKIN is developed for sparse sequencing data, it is also applicable to high-quality genotyping data, for which our estimators reduce to the PC-Relate estimators [21]. We have implemented SEEKIN in an efficient multithreading program, which is publically available at https://github.com/chaolongwang/SEEKIN/.

## Materials and methods

### Genotype calling strategies for shallow sequencing data

A typical genotype calling pipeline involves SNP discovery and genotype inference. In this study, we skipped the SNP discovery step by focusing on biallelic autosomal SNPs that have MAF>0.05 in the 1000 Genomes Project Phase 3 (1KG3) dataset [28]. Given BAM files of *N* individuals, we computed genotype likelihoods across the 1KG3 SNPs using the *mpileup* option in samtools, after filtering reads with mapping quality <30 and base quality <20 [47]. Based on genotype likelihoods, we used three different strategies to generate genotype call sets for downstream analyses. In the first strategy, we used the default settings of bcftools to call genotypes without using any LD information [48]. We set to missing at genotype entries with no read support and filtered SNPs with quality score QUAL<30 or MAF<0.05. In the second strategy, we used BEAGLE (v4.1) to call genotypes by taking genotype likelihoods as the inputs (using the *gl* option) [45]. This strategy leverages the LD information shared among *N* study individuals to improve calling accuracy. In the third strategy, we included 5,008 haplotypes from 1KG3 as the external reference for BEAGLE to improve phasing and genotyping accuracy. We chose BEAGLE because most other imputation programs take genotypes as the input without accounting for genotype uncertainty associated with shallow sequencing data. We set *niterations = 0* in BEAGLE to use its v4.0 phasing algorithm because we found that the genotype probabilities produced by the new algorithm in BEAGLE v4.1 were not well calibrated for shallow sequencing data. For the BEAGLE call sets, we filtered SNPs with dosage r^2^<0.5 or MAF<0.05.

### The SEEKIN method

We propose kinship estimators for shallow sequencing data based on the imputed dosage (i.e., expected genotypic value given the posterior genotype probabilities) and the estimated dosage r^2^ at each SNP, both of which are obtained from BEAGLE. We first describe the relationship between imputed dosages and true genotypes, and then derive kinship estimators for homogeneous samples and for samples with population structure and admixture.

### Relationship between imputed dosages and true genotypes

Suppose *N* individuals from a population are genotyped at *M* biallelic SNPs. Let *G*_*im*_ = 0, 1 or 2 denote the copies of the alternative allele at the *m*^*th*^ SNP of the *i*^*th*^ individual. The expected value for *G*_*im*_ is E(*G*_*im*_) = 2*p*_*m*_ for all *i* = 1,2,…,*N* where *p*_*m*_ is the population allele frequency at the *m*^*th*^ SNP. For commonly used genotype imputation programs, Hu *et al*. [46] derived the expectation of the imputed dosage ${\tilde{G}}_{im}$ given true genotype *G*_*im*_ and the mean genotype ${\bar{G}}_{Rm}$ in the imputation reference panel as

$$E\left({\tilde{G}}_{im}|G_{im},{\bar{G}}_{Rm}\right)=(1-r_{m}^{2}){\bar{G}}_{Rm}+r_{m}^{2}G_{im},$$ (1)

where $r_{m}^{2}$ is the squared correlation between the true genotypes and the imputed dosages at the *m*^*th*^ SNP. Under iterated expectations for Eq (1), the mean of imputed dosage is

$$2{\tilde{p}}_{m}=E\left({\tilde{G}}_{im}|{\bar{G}}_{Rm}\right)=(1-r_{m}^{2}){\bar{G}}_{Rm}+2r_{m}^{2}p_{m}.$$ (2)

Note that $r_{m}^{2}$ can be estimated without knowing the true genotypes and is widely used to measure imputation accuracy [42,43]. We let $\hat{r_{m}^{2}}$ denote the estimate of $r_{m}^{2}$ throughout the rest of the paper.

### Kinship estimators for homogeneous samples

To estimate kinship coefficient *ϕ*_*ij*_ between individuals *i* and *j* using genotypes, Yang *et al*. [5] proposed the genetic relationship estimator:

$$2{\hat{\phi }}_{ij}=\frac{1}{\left|S_{ij}\right|}\sum_{m\in S_{ij}}^{}2{\hat{\phi }}_{ijm}=\frac{1}{\left|S_{ij}\right|}\sum_{m\in S_{ij}}^{}\frac{\left(G_{im}-2p_{m})(G_{jm}-2p_{m}\right)}{2p_{m}(1-p_{m})},$$ (3)

where *S*_*ij*_ is the set of SNPs in the sample with genotypic information for both individuals, and |*S*_*ij*_| is the number of SNPs in this set. Assuming independence across loci, ${\hat{\phi }}_{ij}$ is a consistent estimator of *ϕ*_*ij*_ with |*S*_*ij*_|→∞ [18]. The precision of ${\hat{\phi }}_{ij}$ given in Eq (3) can be improved by averaging over more loci when high quality genotypes are available. For shallow sequencing data, however, a direct substitution of the imputed values $\left({\tilde{G}}_{im},{\tilde{G}}_{jm}\right)$ for (*G*_*im*_,*G*_*jm*_) in Eq (3) could lead to bias in kinship estimation when ignoring the genotype uncertainty. Given Eqs (1) and (2), we propose the following kinship estimator at the *m*^*th*^ SNP:

$$2{\tilde{\phi }}_{ijm}=\frac{\left({\tilde{G}}_{im}-2{\tilde{p}}_{m}\right)\left({\tilde{G}}_{jm}-2{\tilde{p}}_{m}\right)}{2{\tilde{p}}_{m}\left(1-{\tilde{p}}_{m}\right){\left(\hat{r_{m}^{2}}\right)}^{2}},i\neq j,$$ (4)

where ${\tilde{p}}_{m}$ is defined by the first equity of Eq (2) and can be estimated as $\frac{1}{2N}\sum_{i=1}^{N}{\tilde{G}}_{im}$. Based on Eq (2), we further have $p_{m}={\tilde{p}}_{m}-\left({\bar{G}}_{Rm}-2{\tilde{p}}_{m}\right)\left(1-\hat{r_{m}^{2}}\right)/\hat{r_{m}^{2}}$. Because $\left({\bar{G}}_{Rm}-2{\tilde{p}}_{m}\right)\left(1-\hat{r_{m}^{2}}\right)/\hat{r_{m}^{2}}$ is small when the reference panel has similar allele frequency as the imputed samples or when $\hat{r_{m}^{2}}$ is close to 1, we assume $p_{m}={\tilde{p}}_{m}$ unless otherwise noted. Therefore, the main difference between ${\tilde{\phi }}_{ijm}$ and ${\hat{\phi }}_{ijm}$ in Eq (3) is a scaling factor of ${\left(\hat{r_{m}^{2}}\right)}^{2}$ in the denominator, reflecting the observation that the imputed dosages have smaller variance than the true genotypes [46]. When $\hat{r_{m}^{2}}$ goes to 0 for a poorly imputed SNP, the numerator of ${\tilde{\phi }}_{ijm}$ also goes to 0 because all individuals are imputed as ${\bar{G}}_{Rm}$ based on Eq (1), but the expectation of ${\tilde{\phi }}_{ijm}$ remains the same. We show in **S1 Text** that ${\tilde{\phi }}_{ijm}$ share the same expectation with ${\hat{\phi }}_{ijm}$ under the assumption that the residuals of Eq (1) for two different individuals *i* and *j* are independent. When the true genotypes are observed, we have $\left({\tilde{G}}_{im},{\tilde{G}}_{jm})=(G_{im},G_{jm}\right)$ and $\hat{r_{m}^{2}}$ = 1 so that ${\tilde{\phi }}_{ijm}$ reduces to ${\hat{\phi }}_{ijm}$.

We also propose the following estimator of self-kinship coefficient at the *m*^*th*^ SNP:

$$2{\tilde{\phi }}_{iim}=\frac{{\left({\tilde{G}}_{im}-2{\tilde{p}}_{m}\right)}^{2}}{2{\tilde{p}}_{m}\left(1-{\tilde{p}}_{m}\right)\hat{r_{m}^{2}}}.$$ (5)

We show in the **S1 Text** that ${\tilde{\phi }}_{iim}$ has the same expectation as ${\hat{\phi }}_{iim}$ and is an unbiased estimator for (1+*f*_*i*_)/2, where *f*_*i*_ is the inbreeding coefficient of the *i*^*th*^ individual.

In practice, to obtain a genome-wide relationship between individuals *i* and *j*, we combine ${\tilde{\phi }}_{ijm}$ across SNPs using a weighted average:

$${\tilde{\phi }}_{ij}=\frac{\sum_{m}w_{m}{\tilde{\phi }}_{ijm}}{\sum_{m}w_{m}}.$$ (6)

Specific choices of weights *w*_*m*_ generally affect the precision of the estimator but not its expectation. A typical choice is the inverse-variance weighting scheme, which minimizes the sampling variability. We show in **S1 Text** that the variance of ${\tilde{\phi }}_{ijm}$ is inversely proportional to ${\left(\hat{r_{m}^{2}}\right)}^{2}$ when individuals *i* and *j* are unrelated. Furthermore, it has been suggested that down-weighting low-frequency variants can lead to more stable estimation when aggregating information across SNPs [21,49]. Therefore, we propose $w_{m}=2{\tilde{p}}_{m}\left(1-{\tilde{p}}_{m}\right){\left(\hat{r_{m}^{2}}\right)}^{2}$, which intuitively down weighs SNPs of poor imputation quality or of low MAF. Under this weighting scheme, our genome-wide kinship estimator for homogenous samples is

$$2{\tilde{\phi }}_{ij}=\{\begin{array}{c} \frac{\sum_{m}\left({\tilde{G}}_{im}-2{\tilde{p}}_{m}\right)\left({\tilde{G}}_{jm}-2{\tilde{p}}_{m}\right)}{\sum_{m}2{\tilde{p}}_{m}\left(1-{\tilde{p}}_{m}\right){\left(\hat{r_{m}^{2}}\right)}^{2}},i\neq j\\ \frac{\sum_{m}{\left({\tilde{G}}_{im}-2{\tilde{p}}_{m}\right)}^{2}\hat{r_{m}^{2}}}{\sum_{m}2{\tilde{p}}_{m}\left(1-{\tilde{p}}_{m}\right){\left(\hat{r_{m}^{2}}\right)}^{2}},i=j \end{array}.$$ (7)

We denote ${\tilde{\phi }}_{ij}$ in Eq (7) as the SEEKIN-hom estimator.

### Kinship estimators for structured and admixed samples

In the presence of population structure and admixture, the population allele frequency *p*_*m*_ is no longer able to reflect distinct ancestry backgrounds of the individuals. Several existing methods replace population allele frequency *p*_*m*_ with individual-specific allele frequency *p*_*im*_, which is the expected allele frequency given the ancestry of individual *i* [20–22]. For example, the PC-Relate method uses the following estimator:

$$2{\hat{\phi }}_{ij}=\frac{\sum_{m}\left(G_{im}-2p_{im}\right)\left(G_{jm}-2p_{jm}\right)}{\sum_{m}2\sqrt{p_{im}\left(1-p_{im}\right)p_{jm}\left(1-p_{jm}\right)}},$$ (8)

where the individual-specific allele frequencies *p*_*im*_ and *p*_*jm*_ are estimated using linear predictors of top PCs [21,25]. Other methods, including REAP [20] and RelateAdmix [22], derive individual-specific allele frequencies from model-based ancestry estimation programs such as ADMIXTURE [23]. However, neither PCA nor ADMIXTURE can be applied directly to sparse sequencing data. We propose using LASER [38,39], a method that we previously developed for both shallow sequencing and genotyping data, to estimate the top PCs of each study individual in a reference ancestry space. The estimated PCs can be used to predict individual-specific allele frequencies.

Briefly, we first apply PCA on genotyping data of a set of reference individuals to construct an ancestry space using the top *K* PCs, recorded as **V** = [**V**^1^,…,**V**^K^]. Let **G**_*m*_ be a column vector of genotypes at the *m*^*th*^ SNP for the reference individuals. We obtain the least squares solution ${\hat{\beta }}_{m}=\left({\hat{\beta }}_{m0},\ldots ,{\hat{\beta }}_{mK}\right)$ of the linear model E(**G**_**m**_|**V**) = [**1**,**V**]**β**_*m*_ for each SNP. For each sequenced individual *i*, we use LASER to estimate the PC coordinates in the reference ancestry space, denoted as ${\hat{v}}_{i}=\left({\hat{v}}_{i1},\ldots ,{\hat{v}}_{iK}\right)$, in which ${\hat{v}}_{ik}$ is the coordinate of the *k*^*th*^ PC [39]. Similar to PC-Relate [21], we can estimate the allele frequency for individual *i* at the *m*^*th*^ SNP as ${\hat{p}}_{im}=\frac{1}{2}\left({\hat{\beta }}_{m0}+\sum_{k=1}^{K}{\hat{\beta }}_{mk}{\hat{v}}_{ik}\right)$. To avoid out of boundary values, we force ${\hat{p}}_{im}$ to be 0.001 or 0.999 when ${\hat{p}}_{im}<0.001$ or ${\hat{p}}_{im}>0.999$, respectively.

With the estimated individual-specific allele frequencies, we propose the following kinship estimator at the *m*^*th*^ SNP for samples with population structure and admixture:

$$2{\tilde{\phi }}_{ijm}=\frac{\left({\tilde{G}}_{im}-2{\tilde{u}}_{im}\right)\left({\tilde{G}}_{jm}-2{\tilde{u}}_{jm}\right)}{2\sqrt{{\hat{p}}_{im}\left(1-{\hat{p}}_{im}\right){\hat{p}}_{jm}\left(1-{\hat{p}}_{jm}\right)}{\left(\hat{r_{m}^{2}}\right)}^{2}},i\neq j,$$ (9)

where ${\tilde{u}}_{im}={\tilde{p}}_{m}+\hat{r_{m}^{2}}\left({\hat{p}}_{im}-{\hat{p}}_{m}\right)$ and ${\hat{p}}_{m}=\frac{1}{N}\sum_{i}{\hat{p}}_{im}$.

Analogous to Eq (5), the self-kinship coefficient at the *m*^*th*^ SNP can be estimated as:

$$2{\tilde{\phi }}_{iim}=\frac{{\left({\tilde{G}}_{im}-2{\tilde{u}}_{im}^{*}\right)}^{2}}{2{\hat{p}}_{im}\left(1-{\hat{p}}_{im}\right)\hat{r_{m}^{2}}},$$ (10)

where ${\tilde{u}}_{im}^{*}={\tilde{p}}_{m}+\sqrt{\hat{r_{m}^{2}}}\left({\hat{p}}_{im}-{\hat{p}}_{m}\right)$. The terms ${\tilde{u}}_{im}^{}$ and ${\tilde{u}}_{im}^{*}$ can be interpreted as the adjusted individual-specific allele frequencies that account for the imputation accuracy and the shift of allele frequency from the sample average ${\tilde{p}}_{m}$ due to individual ancestry background. Intuitively, the shift should be proportional to $\left({\hat{p}}_{im}-{\hat{p}}_{m}\right)$, reflecting the deviation in allele frequency of an individual from the sample mean. The scaling factors of $\hat{r_{m}^{2}}$ in ${\tilde{u}}_{im}$ and $\sqrt{\hat{r_{m}^{2}}}$ in ${\tilde{u}}_{im}^{*}$ are chosen such that our proposed estimators in Eqs (9) and (10) have the same expectations as the PC-Relate estimator in Eq (8) when individual-specific allele frequencies are accurately estimated (**S1 Text**).

To combine information across genome-wide SNPs, we use the same weighting scheme as the case for the homogeneous samples (Eq 7) but replace population allele frequencies with individual-specific allele frequencies, i.e. $w_{m}=2\sqrt{{\hat{p}}_{im}\left(1-{\hat{p}}_{im}\right){\hat{p}}_{jm}\left(1-{\hat{p}}_{jm}\right)}{\left(\hat{r_{m}^{2}}\right)}^{2}$. Therefore, our proposed kinship estimator for samples with population structure and admixture is

$$2{\tilde{\phi }}_{ij}=\{\begin{array}{c} \frac{\sum_{m}\left({\tilde{G}}_{im}-2{\tilde{u}}_{im}\right)\left({\tilde{G}}_{jm}-2{\tilde{u}}_{jm}\right)}{\sum_{m}2\sqrt{{\hat{p}}_{im}\left(1-{\hat{p}}_{im}\right){\hat{p}}_{jm}\left(1-{\hat{p}}_{jm}\right)}{\left(\hat{r_{m}^{2}}\right)}^{2}},\;i\neq j\\ \frac{\sum_{m}{\left({\tilde{G}}_{im}-2{\tilde{u}}_{im}^{*}\right)}^{2}\hat{r_{m}^{2}}}{\sum_{m}2{\hat{p}}_{im}\left(1-{\hat{p}}_{im}\right){\left(\hat{r_{m}^{2}}\right)}^{2}},i=j \end{array}$$ (11)

When all variants are genotyped or well imputed ($\hat{r_{m}^{2}}\to 1$), we have ${\tilde{p}}_{m}\approx {\hat{p}}_{m}$ and ${\tilde{u}}_{im}\approx {\tilde{u}}_{im}^{*}\approx {\hat{p}}_{im}$ for *m* = 1,2,…,*M*. Our estimator ${\tilde{\phi }}_{ij}$ reduces to the PC-Relate estimator ${\hat{\phi }}_{ij}$ (Eq 8) except that our individual-specific allele frequencies are estimated based on coordinates derived from LASER instead of the PCAiR method [25]. We denote ${\tilde{\phi }}_{ij}$ in Eq (11) as the SEEKIN-het estimator.

### Software implementation

We implemented our SEEKIN estimators into a multithreaded C++ program. The program accepts input files in a standard compressed VCF format. The genotype VCF file can be obtained from BEAGLE, which include genotypes, imputed dosages, and $\hat{r_{m}^{2}}$ for all SNPs. For the SEEKIN-het estimator, SEEKIN requires an additional VCF file that stores the individual-specific allele frequencies. Our program includes a data preparation module to generate the individual-specific allele frequency file and a main module to compute kinship coefficients. To balance computational speed and memory usage, the main module adopts a “single producer/consumer” design pattern (**S1 Fig**). Briefly, a single-threading “producer” job scans the input files, extracts required information for each SNP, and packs into a data block for every *L* SNPs. Concurrently, a “consumer” job takes the data blocks one by one and performs computation. We simultaneously compute all elements in a kinship matrix of *N* individuals by adopting matrix representations of the estimators in Eqs (7) and (11). Our implementation uses the Armadillo C++ library [50], which provides multithreading and highly efficient matrix computation. The required memory of SEEKIN scales as O(*N*^*2*^*L*). The block size *L* can be specified by users according to the available computational resource, making our software scalable to large datasets.

### Sequencing and genotyping data from the Singapore Living Biobank Project

The Singapore Living Biobank is a collection of healthy population-based Chinese and Malay individuals, for the purpose of phenotype recall study of high-impact variant carriers. These individuals are sampled from two studies: Multi-Ethnic Cohort (MEC), and the Singapore Health 2012 (SH2012). The MEC is a population-based cohort initiated in 2007 to investigate the genetic and lifestyle factors that affect the risk of developing chronic diseases such as diabetes and cardiovascular outcomes in the three ethnic groups (Chinese, Malay, and Indian). The SH2012 study is a population-based cross-sectional survey conducted in Singapore between 2012 and 2013, with over-sampling of Malays and Indians [51]. Participants in MEC and SH2012 completed a similar set of questionnaire components, health examination, and biochemisty panels. Description of the MEC and SH2012 studies can be found at http://blog.nus.edu.sg/sphs/. The National University of Singapore Institutional Review Board approved the Living Biobank Project (Approval No.: NUS 2585). All participants provided written informed consent.

In total, 1,299 self-reported Chinese and 1,229 self-reported Malays were whole-exome sequenced on the Illumina HiSeq2000 platform (125bp paired end). The exonic regions were captured using the Nimblegen SeqCap EZ Exome v3 kits. We aligned sequence reads to the human reference genome (GRCh37) using BWA-MEM [52], followed by base quality score recalibration and removal of duplicated reads [53]. The mean depth of raw reads aligned to the target regions was ~32X. After excluding reads with mapping quality score <30 and base quality score <20, the mean sequencing depths across target and off-target regions were ~20X and ~0.75X, respectively. We focused on off-target data in our evaluation of low-coverage settings. In addition, we used samtools [47] to down sample 20% of the off-target data, which was ~0.15X, to mimic a typical off-target coverage in studies that sequence small target regions rather than the whole exome [33,38].

Among the sequenced individuals, we have array genotyping data for 2,452 individuals (Illumina OmniExpress-24). After excluding SNPs with call rate <0.95, HWE P<10^−5^ in either Chinese or Malay, or minor allele frequency (MAF) <0.01, we retained 595,668 autosomal SNPs.

### Inference of population structure and relatedness in the Singapore Living Biobank Project

We jointly analyzed the array genotyping data of 2,452 individuals from the Singapore Living Biobank Project with 268 individuals from the Singapore Genome Variation Project (SGVP) [54]. The SGVP includes 96 Chinese, 89 Malays, and 83 Indians, who were genotyped on Affymetrix 6.0 and Illumina Human1M arrays, totaling 1,141,519 autosomal SNPs with MAF>0.05. Based on 435,314 overlapping SNPs, we estimated the genetic ancestry background of the Living Biobank samples using ADMIXTURE and LASER [23,39], both including the SGVP dataset as reference. For the ADMIXTURE analysis, we used the supervised mode and set the number of clusters *K = 3* because Singapore has three major ethnicity groups. We plotted results from ADMIXTURE using CLUMPAK [55]. The LASER method can analyze either genotypes or sequence reads to infer an individual’s ancestry in a reference ancestry space [39]. We used the default settings of the *trace* program in LASER to place the Living Biobank samples in the ancestry space generated by the first two principal components (PCs) of the SGVP individuals.

We applied PC-Relate [21] to the array genotyping data to estimate both kinship coefficients and the probability of zero IBD sharing. Using the criteria in [18], we identified 736 pairs of close relatedness (≤3^rd^ degree), involving 263 Chinese and 499 Malay individuals. In this paper, we focused on these 762 individuals to evaluate different kinship estimators on low-coverage sequencing data. Because pedigree information was not collected, we used the kinship coefficients estimated by PC-Relate on the array genotyping data as the gold standard for comparison.

### Simulations and estimation of trait heritability

We evaluated the impacts of kinship estimation on downstream analysis of trait heritability based on 762 related individuals from the Singapore Living Biobank Project. We first simulated quantitative traits using a linear mixed model **y** ∼ *N*(0,2**Φ** + **I**), where **Φ** is the kinship matrix estimated by PC-Relate on the GWAS array data and **I** is the identity matrix. The simulated traits have heritability *h*^2^ = 0.5 under this model. We then estimated heritability using different kinship matrices derived from sequencing data within WES target regions or across both target and off-target regions using either SEEKIN or PC-Relate. For the off-target regions, we experimented with both the original data (~0.75X) and the down sampled data (~0.15X). Heritability estimation was performed using the restricted maximum likelihood (REML) method in the GEMMA software [2].

We also compared heritability estimation for 10 metabolic traits using GWAS array data, WES target data, or WES target and off-target data. These traits include body-mass index (BMI), waist-to-hip ratio (WHR), systolic blood pressure (SBP), diastolic blood pressure (DBP), total cholesterol (TC), low-density lipoprotein (LDL), high-density lipoprotein (HDL), triglycerides (TG), fasting blood glucose (FBG) and hemoglobin A1C (HbA1C). We log-transformed TG to reduce the skewness of its distribution. For each trait, we removed outliers that are more than 5 standard deviations from the mean. We used the REML method in GEMMA to estimate heritability for each trait, adjusting for age, age^2^, sex, and the first two ancestry PCs. The ancestry PCs were derived from LASER using array genotypes and the SGVP reference panel [39].

## Results

### Population structure and relatedness in the Singapore Living Biobank Project

**Fig 1:**
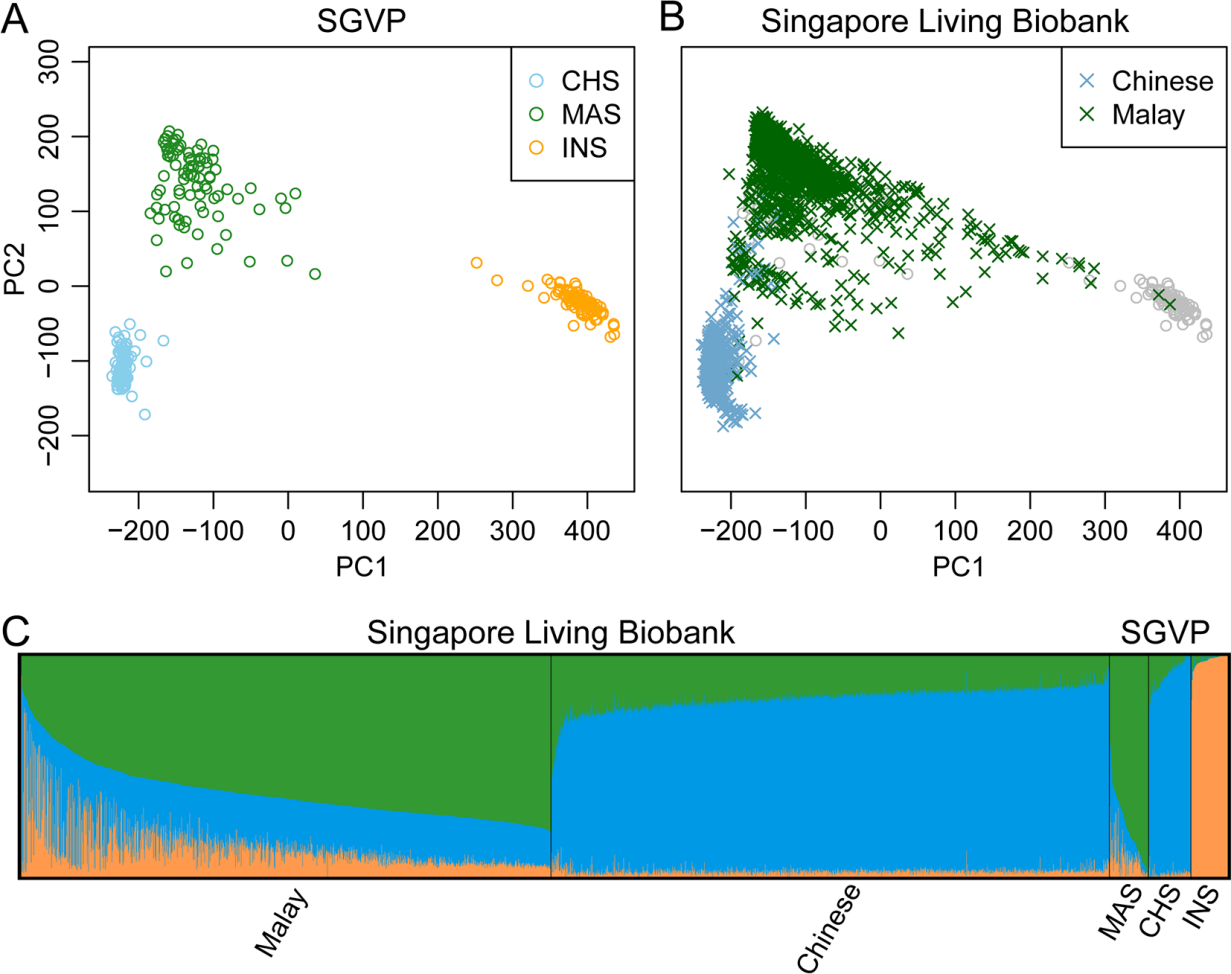
Population structure of 2,452 individuals in the Singapore Living Biobank Project. (A) Reference ancestry space derived from PCA on the genotypes of Chinese (CHS), Malays (MAS) and Indians (INS) from SGVP. (B) Estimated ancestry in the SGVP reference space based on LASER analysis. Colored symbols represent study individuals of self-reported Chinese and Malays. Grey symbols represent the SGVP reference individuals. (C) Estimated admixture proportion based on supervised ADMIXTURE analysis with the SGVP data as the reference. We specified K = 3 clusters in the ADMIXTURE analysis, which represent Chinese (blue), Malay (green), and Indian (orange) ancestry components.

Three major ethnic groups, Chinese, Malay and Indian, contribute to ~97% of the population in Singapore. Using genotypes across 435,314 SNPs, we compared the ancestry backgrounds of 2,452 individuals in the Singapore Living Biobank with 268 individuals previously reported by the Singapore Genome Variation Project (SGVP) [54]. The SGVP samples were selected on the basis that all four grandparents belong to the same ethnic group and thus were less likely to be admixed [54]. Based on the first two PCs derived from the LASER analysis (**Fig 1A and 1B**), self-reported Chinese from the Living Biobank Project tightly cluster with each other and with the SGVP Chinese, expect for a few outliers. In contrast, self-reported Malays appear to be more heterogeneous, with many individuals spreading between different ethnicity groups in the SGVP, indicating a high level of admixture among self-reported Malays from the Living Biobank Project. Such observations were confirmed by the ADMIXTURE analysis [23]. Self-reported Malays had ~25% Chinese ancestry component and ~13% Indian ancestry component, and the variation of admixture proportions is large across individuals (**Fig 1C**). Compared to Malays, self-reported Chinese are more homogeneous with ~3% Indian component and ~19% Malay component. The moderate level of shared ancestry component between most Chinese and Malays may reflect recent split between these two populations in addition to potential admixture events.

**Fig 2:**
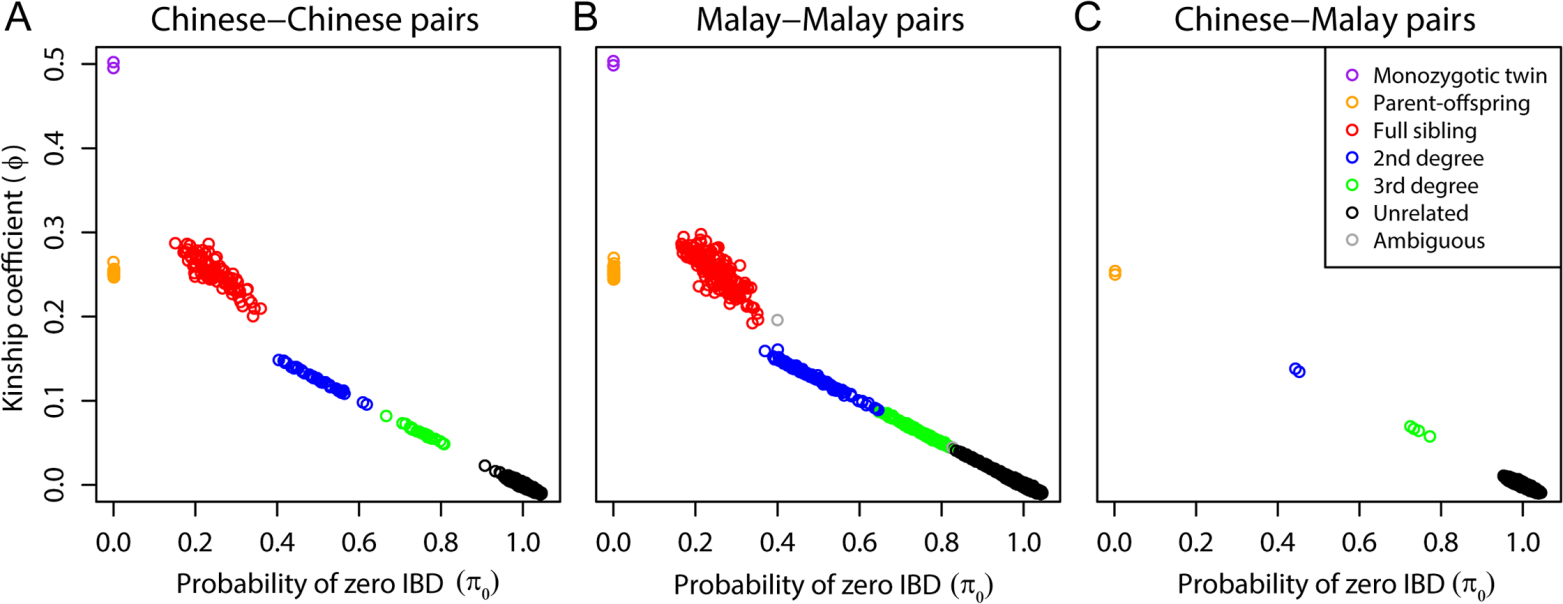
Cryptic relatedness among 2,452 individuals in the Singapore Living Biobank Project. We estimated kinship coefficient *ϕ* and the proportion of zero-IBD-sharing *π*_0_ for each pair of individuals using PC-Relate. Relatedness types were determined using the inference criteria of *ϕ* and *π*_0_ given by [18]. An ambiguous relationship was inferred if the criteria of *ϕ* and *π*_0_ were not met simultaneously. (A) Results for pairs of Chinese. (B) Results for pairs of Malays. (C) Results for pairs that consist of a Chinese and a Malay.

Given the presence of population structure and admixture, we used PC-Relate [21] to infer relatedness between the Living Biobank samples (**Fig 2**). Results derived from REAP [20] and RelateAdmix [22] are similar. We classified close relatedness into monozygotic twins (MZ), parent-offspring (PO), full siblings (FS), 2^nd^ degree and 3^rd^ degree based on the estimated kinship coefficient *ϕ* and the probability of zero-IBD-sharing *π*_0_ with thresholds given in [18]. After excluding two pairs with ambiguous relationship (i.e., *ϕ* falls in the range of PO/FS relatedness but *π*_0_ falls in the range of 2^nd^ degree relatedness), we found two MZ, 53 PO, 96 FS, 38 2^nd^ degree and 24 3^rd^ degree pairs of Chinese, and two MZ, 99 PO, 187 FS, 107 2^nd^ degree and 120 3^rd^ degree pairs of Malays. Interestingly, we also identified eight closely related pairs of one Chinese and one Malay, including two PO, and two 2^nd^ degree and four 3^rd^ degree pairs. We further checked the admixture proportion of these eight Chinese-Malay related pairs and found that all of the eight self-reported Chinese have >35% Malay component, much higher than the average level of ~19% in Chinese. These results provide clear genetic evidence of recent admixture between Chinese and Malay populations. In total, 263 Chinese and 499 Malays (~31% of the total sample) were identified to have close relatives in the sample. We used these individuals to form test datasets to evaluate the performance of different kinship estimators in a homogeneous sample that includes only Chinese (N = 254 after excluding nine Chinese with >35% Malay admixture component) and a heterogeneous sample of pooled Chinese and Malays (N = 762).

### Sequence-based estimation of kinship in homogeneous samples

To evaluate performance of kinship estimators based on off-target sequencing data in typical target sequencing experiments, we down sampled from the original WES data to generate a low-coverage sequencing dataset of ~0.15X depth (**Materials and Methods**). Our evaluation of homogeneous estimators was based on 254 related Chinese individuals. We compared our SEEKIN-hom estimator (Eq 7) with existing estimators for homogeneous samples, including lcMLkin [40], GCTA [5], and KING (specifically the homogeneous estimator, KING-hom) [18].

**Fig 3:**
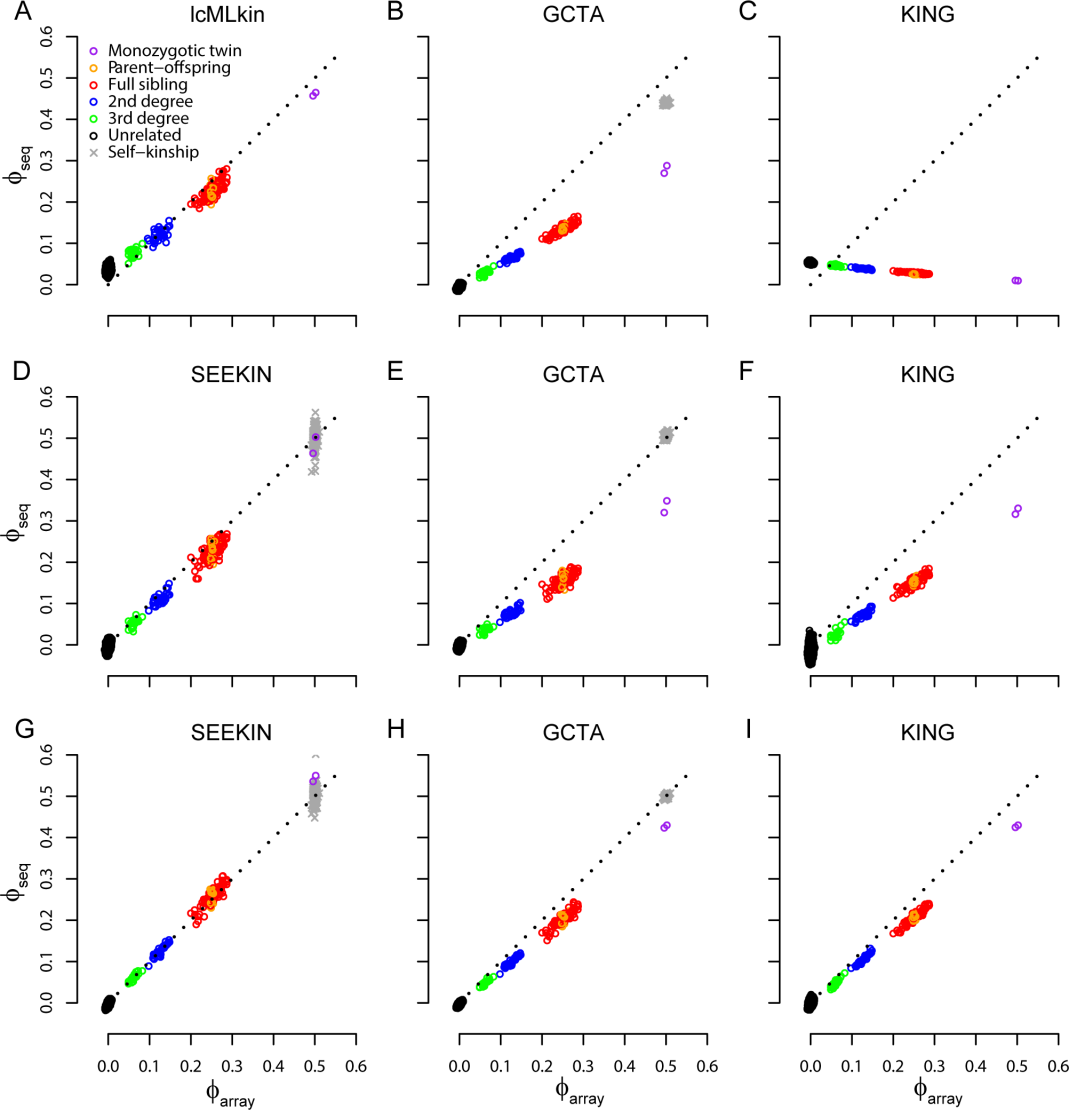
Performance of homogeneous kinship estimators in ~0.15X sequencing data of 254 Chinese. In each panel, we compared sequence-based estimates (*ϕ*_seq_, y-axis) with the array-based estimates from PC-Relate (*ϕ*_array_, x-axis). Colored circles represent kinship coefficients between two individuals and different types of relatedness were determined in Fig 2. Grey crosses represent self-kinship coefficients. We evaluated lcMLkin (A), GCTA (B, E, H), KING (C, F, I), and SEEKIN (D, G) using the bcftools call set (A-C), the BEAGLE call set (D-F), and the BEAGLE+1KG3 call set (G-I). Note that lcMLkin and KING do not estimate self-kinship coefficients.

First, we used bcftools to call genotypes for these 254 individuals without using LD information [48]. Even though 1,541,541 SNPs with MAF≥0.05 were identified, the number of overlapping SNPs between any pair of individuals was only ~46,379 due to large amounts of missing data. Both GCTA and KING performed poorly with strong downward bias in comparison to the gold standard based on array genotyping data (**Fig 3A**; **Table 1**). Due to high computational demands of lcMLkin, we had to trim the full dataset to one SNP in every 20kb genomic region, resulting in 106,247 independent SNPs for the lcMLkin analysis. By modeling genotype uncertainty, lcMLkin performed better than GCTA and KING, but still systematically underestimated kinship for PO/FS pairs by ~0.026 and overestimated kinship for unrelated pairs by ~0.035.

**Table 1:** Performance of homogeneous kinship estimators in ~0.15X sequencing data of 254 Chinese.

Call set	Method	Unrelated (31,925 pairs)		3^rd^ degree (22 pairs)		2^nd^ degree (36 pairs)		PO/FS (146 pairs)		Self-kinship (254 individuals)	
		RMSE	BIAS	RMSE	BIAS	RMSE	BIAS	RMSE	BIAS	RMSE	BIAS
Bcftools	lcMLkin	0.035	0.035	0.019	0.016*	0.013*	-0.004*	0.028*	-0.026*	—	—
	GCTA	0.007*	-0.006*	0.034	-0.033	0.062	-0.061	0.116	-0.116	0.123	-0.122
	KING	0.053	0.053	0.018*	-0.016*	0.088	-0.087	0.225	-0.225	—	—
BEAGLE	SEEKIN	0.007	-0.004	0.012*	-0.009*	0.018*	-0.016*	0.028*	-0.023*	0.043	-0.003*
	GCTA	0.005*	-0.003*	0.027	-0.026	0.050	-0.049	0.094	-0.093	0.014*	0.011
	KING	0.017	-0.014	0.036	-0.036	0.054	-0.054	0.099	-0.099	—	—
BEAGLE+1KG3	SEEKIN	0.005	-0.004	0.004*	-0.001*	0.006*	-0.001*	0.013*	0.008*	0.032	0.002*
	GCTA	0.004*	-0.003	0.014	-0.014	0.027	-0.027	0.047	-0.046	0.007*	-0.009
	KING	0.005	-0.002*	0.014	-0.013	0.022	-0.022	0.044	-0.043	—	—

RMSE is the root mean squared error and BIAS is defined as the mean difference to the array-based estimates from PC-Relate for each type of relatedness. Negative values of BIAS suggest underestimation for results based on sparse sequencing data and vice versa.

* Smallest magnitude of RMSE or BIAS in each call set and each type of relatedness.

Next, we used BEAGLE without external reference data to call genotypes [42]. This approach uses shared LD information among the individuals to both improve genotype accuracy and impute missing data. After excluding SNPs with MAF<0.05 or r^2^<0.5, the remaining set includes 68,785 SNPs with no missing genotypes. The lcMLkin method cannot be applied to this call set because lcMLkin requires genotype likelihoods, which are not available in the LD-based call set generated by BEAGLE. GCTA and KING had improved performance using this call set but still systematically underestimated kinship coefficients (**Fig 3B**; **Table 1**). In comparison, our SEEKIN estimator largely reduced the bias by accounting for genotype uncertainty intrinsic to low-coverage sequencing data. For example, the mean downward bias of the estimated kinship coefficients for PO/FS pairs is 0.023 for SEEKIN, much lower than 0.093 for GCTA and 0.099 for KING. Similar observations hold for other types of relatedness that SEEKIN has the lowest bias and RMSE, except for the unrelated pairs in which GCTA is slightly better than SEEKIN (**Table 1**). For self-kinship coefficients, estimates derived from SEEKIN have little bias as we expect, but the RMSE is higher for SEEKIN (0.043) than for GCTA (0.014). KING does not estimate self-kinship coefficients. It seems counterintuitive that GCTA substantially underestimated kinship coefficients for MZ pairs but performed well in estimating self-kinship coefficients, given that the underlying genotypes are identical for MZ pairs. Our explanation is that at low-coverage setting, the most-likely genotypes in each individual tend to follow a prior assumption of HWE. This is equivalent to assuming a self-kinship of 0.5, close to the truth in human populations with little inbreeding. For SEEKIN, self-kinship estimates have much larger variation than pairwise kinship estimates, which might be due to different amounts of data used in the estimation; self-kinship coefficients were estimated based on data from a single sample, while pairwise kinship coefficients were derived using data from two samples.

By incorporating external haplotypes as the reference panel in BEAGLE, we can substantially improve the genotype calling quality for low-coverage sequencing data [41]. In our call set with the 1KG3 reference panel [28], we retained 4,517,106 SNPs with MAF≥0.05 and r^2^≥0.5, ~66 times more SNPs than the BEAGLE call set without reference. Furthermore, the genotype concordance rate for SNPs overlapping with the array data increased from 0.85 to 0.90. The improved genotype quality led to better performance for all methods (**Fig 3C**; **Table 1**). Nevertheless, GCTA and KING still consistently underestimated kinship coefficients for closely related pairs, while SEEKIN had the smallest empirical bias (almost 0) and RMSE values (~3–4 times smaller than GCTA and KING). All three methods performed similarly for unrelated pairs. The SEEKIN estimation of self-kinship coefficients remained inaccurate (RMSE = 0.032).

We further evaluated accuracy of relationship classification based on the pairwise kinship estimates. Manichaikul et al. [18] proposed a set of classification criteria, in which the ranges of kinship coefficients for PO/FS, 2^nd^ degree, and 3^rd^ degree related pairs are (2^−5/2^, 2^−3/2^), (2^−7/2^, 2^−5/2^), and (2^−9/2^, 2^−7/2^), respectively. We applied the same set of criteria on our kinship estimates to classify relationship. We used the relationship types inferred from array-based kinship estimates as the gold standard (**Fig 2**), and calculated the sensitivity and precision in classifying each relationship type using the sequence-based kinship estimates. Due to more accurate kinship estimates, relationship classification based on SEEKIN outperformed other methods (**S1 Table**). For example, using the BEAGLE+1KG3 call set, SEEKIN achieved perfect sensitivity and precision in classifying PO/FS, 2^nd^, and 3^rd^ degree relationship with only ~0.15X sequencing data, while both GCTA and KING had <96%, <92%, and <63% sensitivity to identify PO/FS, 2^nd^, and 3^rd^ degree relationship, respectively.

We also repeated the evaluation for both kinship estimation and relationship classification using all the off-target sequencing data at ~0.75X without down sampling. While all methods had improved performance compared to using ~0.15X data, kinship estimation using GCTA and KING remained downward biased in all three call sets (**S2 Fig**; **S2 Table**). The sensitivity and precision of relationship classification were highest for the SEEKIN method (**S3 Table**). For the BEAGLE call set, GCTA and KING misclassified >40% of the 2^nd^ degree relatedness as the 3^rd^ degree relatedness due to underestimation of kinship coefficients, while SEEKIN only misclassified ~2.8% of the 2^nd^ degree relatedness. When applied to the 1KG3-guided BEAGLE call set, our SEEKIN method produced kinship estimates almost identical to the gold standard based on array genotyping data (RMSE≤0.007 for all relatedness types). Kinship estimation and relationship classification were also much improved for KING and GCTA. It is worth noting that in this setting, the variation of SEEKIN estimates of self-kinship coefficients was much reduced (RMSE = 0.018, similar to RMSE = 0.015 for GCTA).

### Sequence-based estimation of kinship with population structure and admixture

To evaluate kinship estimators for heterogeneous samples, we pooled all 762 related individuals from the Singapore Living Biobank Project to form test datasets that include Chinese, Malays and admixed individuals. We evaluated our SEEKIN-het estimator (Eq 11) and existing estimators PC-Relate [21], REAP [20], and RelateAdmix [22] at sequencing depth of 0.15X and 0.75X. We used the SGVP dataset [54] as the reference panel in LASER [39] and ADMIXTURE [23] analyses to derive individual ancestry and thereby individual-specific allele frequencies for SEEKIN, REAP and RelateAdmix. Therefore, our analyses were restricted to SNPs overlapping with the SGVP dataset, including PC-Relate which does not require an external ancestry reference panel. We did not compare with homogeneous estimators because they have been shown by previous studies to perform poorly on admixed samples [20–22].

**Fig 4:**
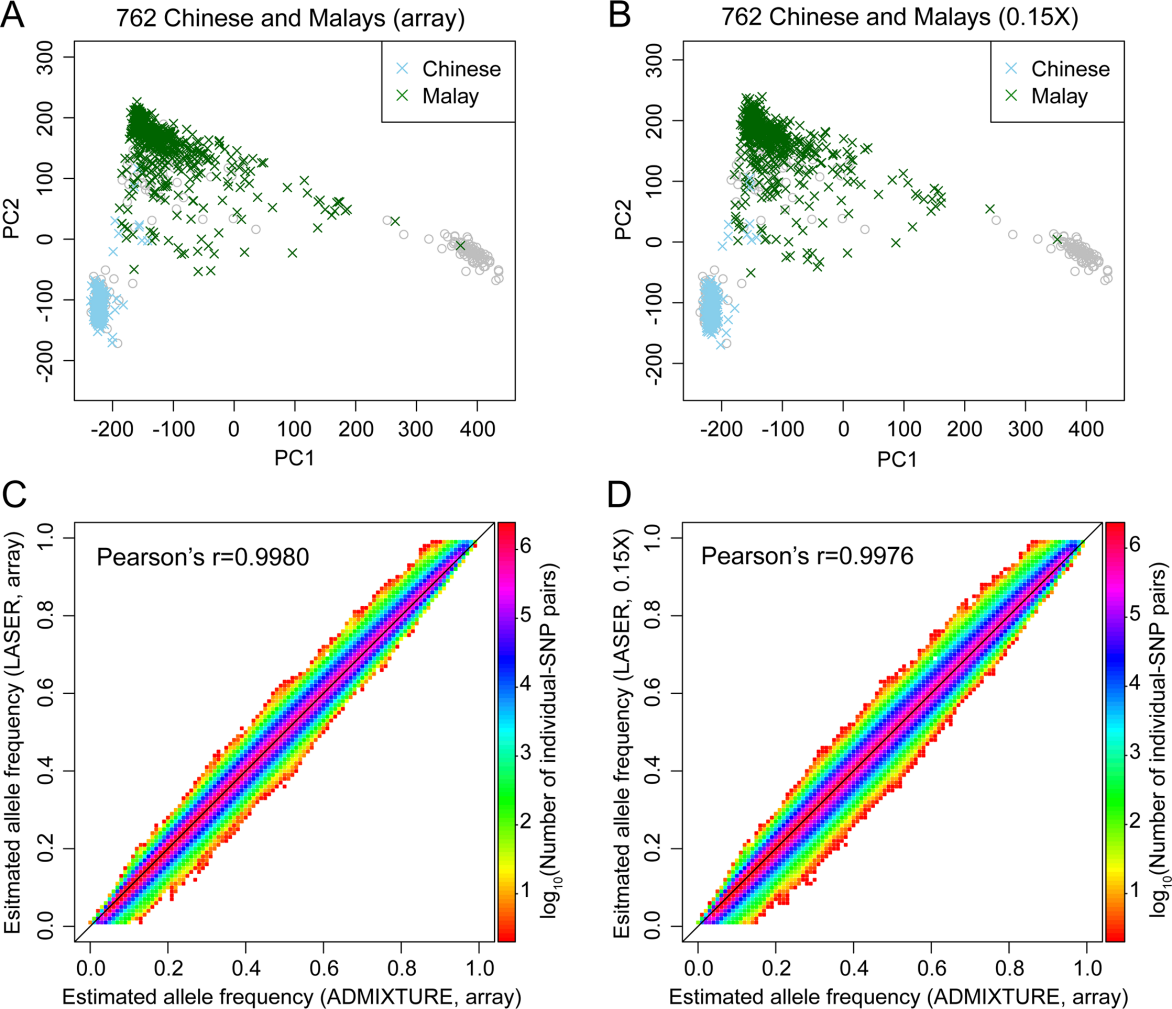
Ancestry and individual-specific allele frequency estimation using array data or ~0.15X sequencing data of 762 Chinese and Malays. (A-B) LASER ancestry estimates based on array genotypes across 435,314 SNPs overlapping with the SGVP reference dataset (A) or ~0.15X sequence reads scattering genome-wide (B). Colored symbols represent study individuals and grey symbols represent the SGVP reference individuals. The Procrustes similarity between (A) and (B) is t_0_ = 0.9976 for 762 study individuals. (C-D) Comparison of individual-specific allele frequencies derived from LASER analysis of either array data (C) or ~0.15X sequencing data (D) to the gold standard based on ADMIXTURE analysis of array data. The two-way allele frequency space is evenly into 100×100 grids and the number of data points within each grid is color-coded according to the logarithmic scale in the color bar. The Pearson correlation is r = 0.9980 across all data points in (C) and is r = 0.9976 across all data points in (D).

Before proceeding to kinship estimation, we evaluated if we could accurately estimate individual-specific allele frequencies for sparsely sequenced samples. First, we confirmed that LASER can produce accurate estimation of top PCs using sparse sequencing data. For 762 Chinese and Malays, the top two PCs in the SGVP ancestry space estimated from 0.15X sequencing data are almost identical to those derived from GWAS array data (Procrustes similarity t_0_ = 0.9976,**Fig 4A and 4B**) [56]. Next, we compared individual-specific allele frequencies predicted by top two LASER PCs from either array data or 0.15X sequencing data with those from ADMIXTURE analysis of array data. Here, we used the individual-specific allele frequencies derived from ADMXITURE as the gold standard, because ADMIXTURE is a rigorous model-based approach with superior performance demonstrated by previous studies [20,22,23]. We showed that using array data, the PC-based individual-specific allele frequencies are highly consistent with those derived from ADMIXTURE (Pearson correlation r = 0.9980,**Fig 4C and 4D**). The correlation dropped slightly to 0.9976 when the PCs were derived from 0.15X sequencing data instead of array data. These results suggests that our approach based on LASER can accurately estimate individual-specific allele frequencies even when the sequencing depth is extremely low.

**Fig 5:**
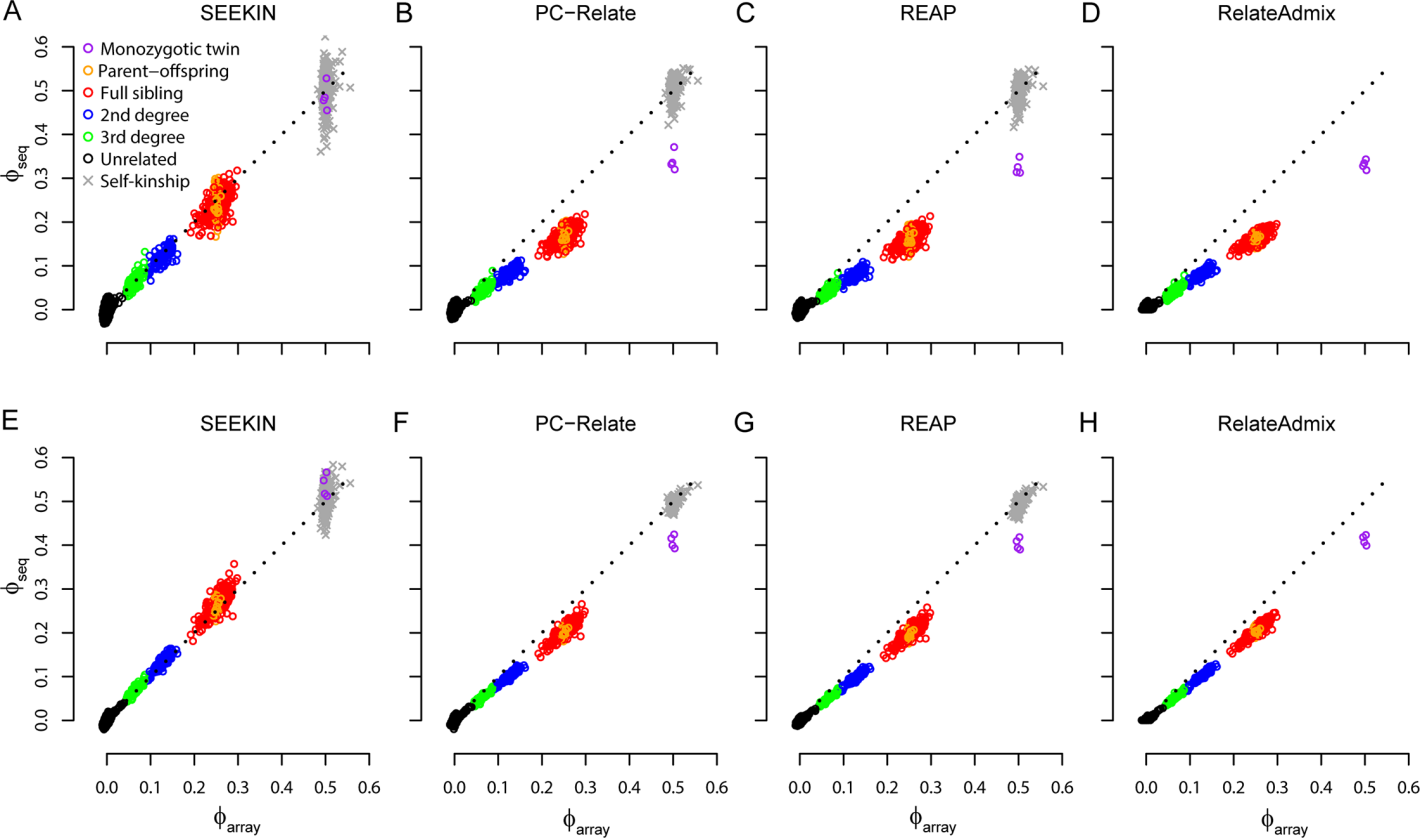
Performance of heterogeneous kinship estimators in ~0.15X sequencing data of 762 Chinese and Malays. In each panel, we compared sequence-based estimates (*ϕ*_seq_, y-axis) with the array-based estimates from PC-Relate (*ϕ*_array_, x-axis). Colored circles represent kinship coefficients between two individuals and different types of relatedness were determined in Fig 2. Grey crosses represent self-kinship coefficients. We evaluated SEEKIN (A, E), PC-Relate (B, F), REAP (C, G), and RelateAdmix (D, H) using the BEAGLE call set (A-D), and the BEAGLE+1KG3 call set (E-H). We only included SNPs overlapping with the SGVP dataset in the analyses, because we used the SGVP dataset as the reference panel to estimate individual-specific allele frequencies for SEEKIN, REAP and RelateAdmix.

For kinship estimation in heterogeneous samples, we only considered the BEAGLE and BEAGLE+1KG3 call sets, because we have shown that LD-based call sets performed much better than the bcftools call set at low-coverage setting (**Figs 3** and **S2**). Without modeling the genotype uncertainty, PC-Relate, REAP, and RelateAdmix underestimated kinship coefficients for related pairs at both 0.15X and 0.75X sequencing depth (**Figs 5** and **S3**; **Tables 2** and **S4**). In contrast, SEEKIN reduced the RMSE by >50% and the empirical bias by >65% for kinship estimates between close relatives. In particular, based on the BEAGLE+1KG3 call set at 0.75X, SEEKIN’s estimates were almost identical to the gold standard based on array data (RMSE≤0.007). SEEKIN performed similarly to existing methods for unrelated pairs. For self-kinship coefficients, SEEKIN estimates had large RMSE, especially at 0.15X, even though the empirical bias was small. The estimates of self-kinship coefficients became more accurate on the BEAGLE+1KG3 call set at 0.75X, where all three methods had similar RMSE (0.018 for SEEKIN and REAP, and 0.017 for PC-Relate), but SEEKIN has the smallest empirical bias (0.002 for SEEKIN, -0.014 for PC-Relate, and -0.017 for REAP). For relationship classification, SEEKIN remained the best among all methods in terms of both sensitivity and precision, regardless of sequencing depth and relationship types (**S5 Table; S6 Table**). Remarkably, SEEKIN achieved >92% precision and >86% sensitivity in classifying 3^rd^ and 2^nd^ degree relatedness based on the BEAGLE call set at 0.15X, while PC-Relate, REAP, and RelateAdmix, had <40% precision and sensitivity. For the BEAGLE+1KG3 call set at 0.15X, SEEKIN had >95% precision and sensitivity in classifying 3^rd^ and 2^nd^ degree relatedness, while the same metrics for the other methods were <90%. Overall, the performance of the SEEKIN-het estimator on heterogeneous samples is similar to that of SEEKIN-hom on homogeneous samples, suggesting that SEEKIN-het effectively accounts for the diverse ancestry background in samples with population structure and admixture.

**Table 2:** Performance of heterogeneous kinship estimators in ~0.15X sequencing data of 762 Chinese and Malays.

Call set	Method	Unrelated (289,205 pairs)		3^rd^ degree (148 pairs)		2^nd^ degree (147pairs)		PO/FS (437 pairs)		Self-kinship (762 individuals)	
		RMSE	BIAS	RMSE	BIAS	RMSE	BIAS	RMSE	BIAS	RMSE	BIAS
BEAGLE	SEEKIN	0.007	-0.002	0.010*	-0.001*	0.014*	-0.007*	0.025*	-0.010*	0.058	0.006
	PC-Relate	0.005	0.000*	0.022	-0.021	0.044	-0.043	0.084	-0.083	0.035	0.018
	REAP	0.004*	-0.001	0.024	-0.023	0.048	-0.048	0.091	-0.090	0.033*	-0.005*
	RelateAdmix	0.004*	0.002	0.023	-0.022	0.046	-0.045	0.088	-0.087	—	—
BEAGLE+1KG3	SEEKIN	0.004	-0.002	0.006*	0.004*	0.009*	0.006*	0.021*	0.015*	0.041	0.018
	PC-Relate	0.002*	0.000*	0.011	-0.011	0.025	-0.024	0.049	-0.048	0.014*	-0.008*
	REAP	0.002*	-0.001	0.015	-0.015	0.030	-0.029	0.054	-0.053	0.020	-0.014
	RelateAdmix	0.002*	0.001	0.013	-0.013	0.026	-0.025	0.048	-0.047	—	—

RMSE is the root mean squared error and BIAS is defined as the mean difference to the array-based estimates from PC-Relate for each type of relatedness. Negative values of BIAS suggest underestimation for results based on sparse sequencing data and vice versa.

* Smallest magnitude of RMSE or BIAS in each call set and each type of relatedness.

### Estimation of kinship and trait heritability for WES data

In this section, we evaluated how SEEKIN can improve kinship estimation in WES studies by incorporating off-target sequencing data, in comparison to the conventional approach that discards off-target data. We analyzed the original WES data of 762 Chinese and Malays, jointly called using BEAGLE with the 1KG3 reference panel across both target and off-target regions. To illustrate the benefits in downstream analyses, we compared heritability estimation based on different estimated kinship matrices for both simulated polygenic traits and 10 metabolic traits.

**Fig 6:**
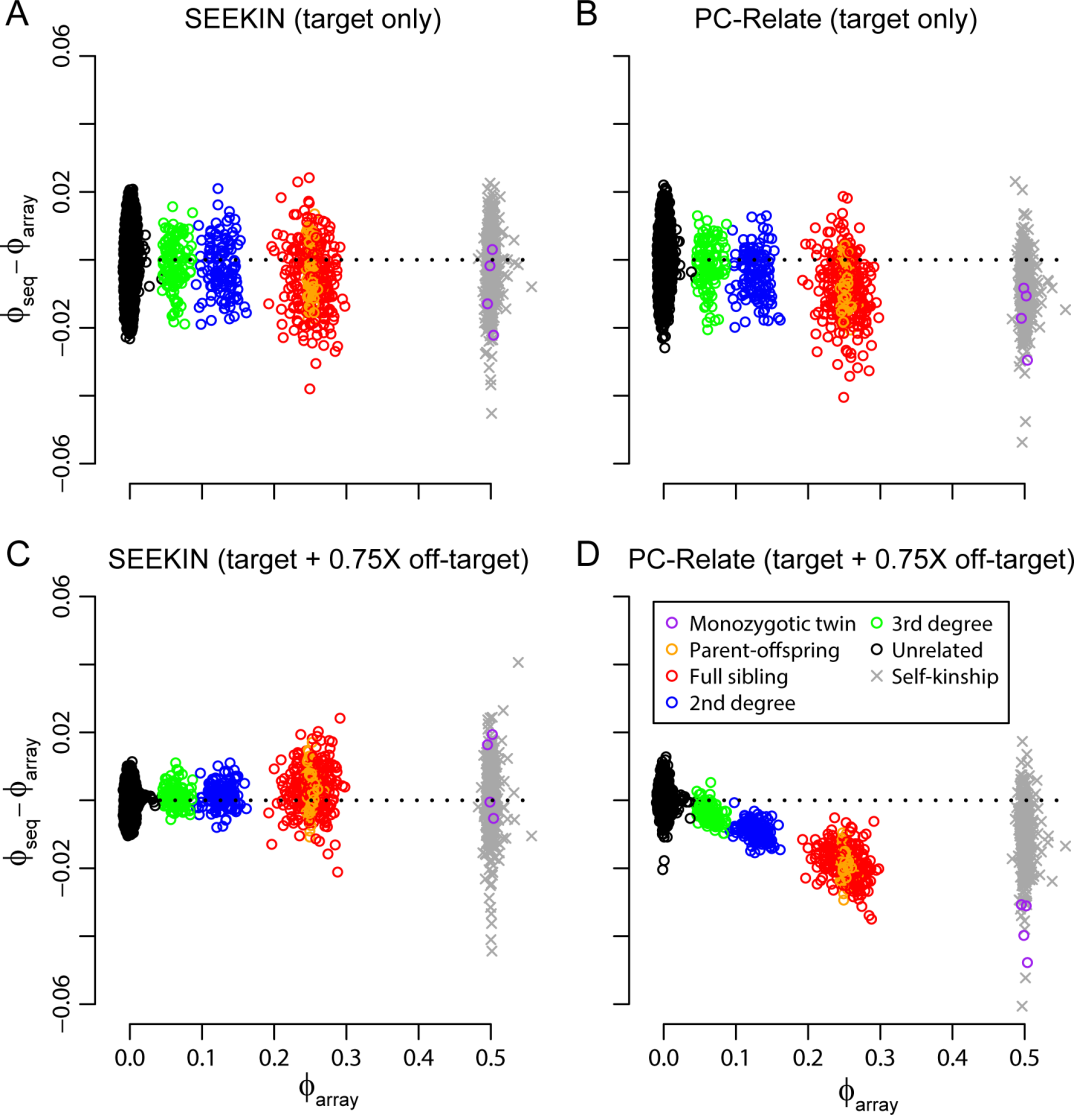
Off-target sequencing data improve kinship estimation in WES of 762 Chinese and Malays. In each panel, we plotted the difference between sequence-based estimates and array-based estimates (*ϕ*_seq_–*ϕ*_array_, y-axis) versus the array-based estimates from PC-Relate (*ϕ*_array_, x-axis). Colored circles represent kinship coefficients between two individuals and different types of relatedness were determined in Fig 2. Grey crosses represent self-kinship coefficients. The analyses were based on the BEAGLE+1KG3 call set at SNPs overlapping with the SGVP dataset. We evaluated SEEKIN (A, C) and PC-Relate (B, D) using 40,824 SNPs within the WES target regions or 1,054,229 SNPs across both target and off-target regions.

**Fig 7:**
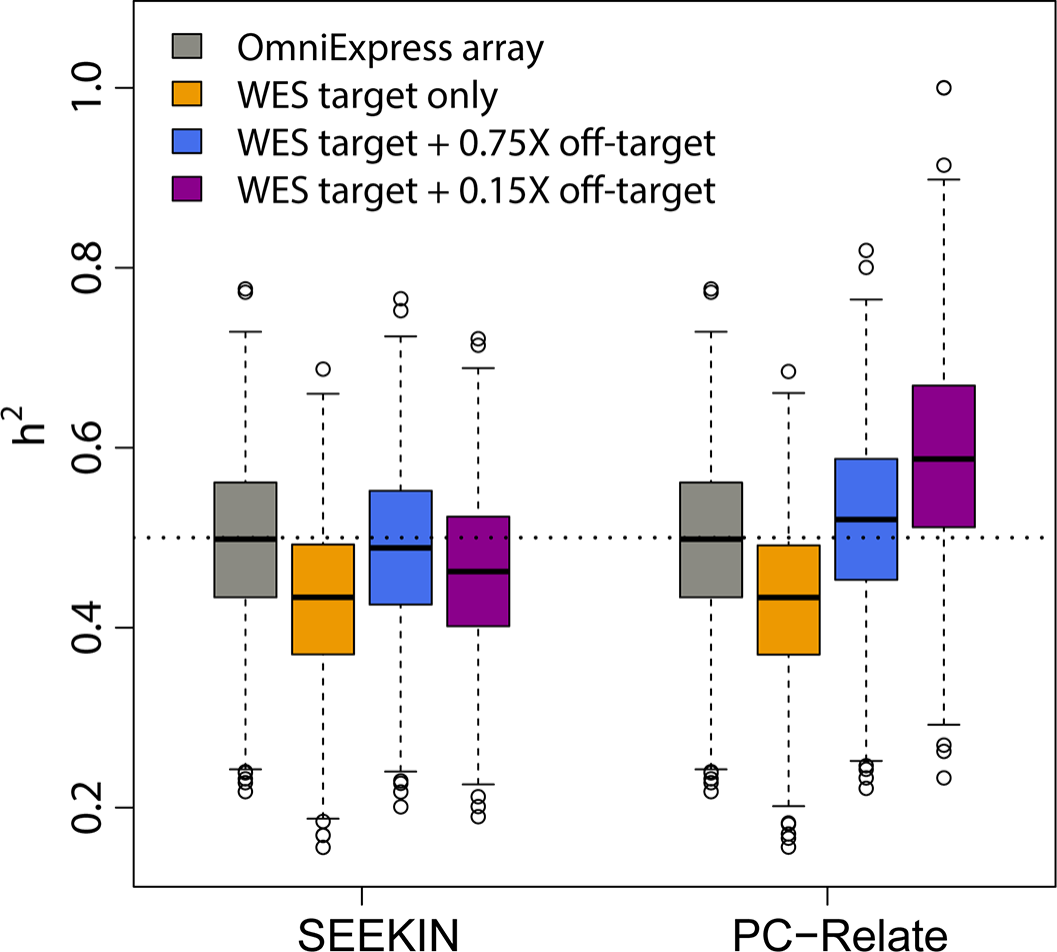
Heritability estimation for simulated traits in 762 Chinese and Malays. We simulated quantitative traits of heritability *h*^2^ = 0.5 using a linear mixed model *Y*∼*N*(0,2**Φ** + ***I***), where **Φ** is the array-based kinship matrix from PC-Relate and ***I*** is the identity matrix. We used the REML method in GEMMA to estimate heritability based on kinship matrices derived from WES data with or without off-target data using SEEKIN or PC-Relate (Fig 6). We also considered a case where the off-target data were down-sampled to ~0.15X but the target data remained the same. Each box represents heritability estimates of 1,000 replicates.

When we focused on target regions, genotypes across 40,824 SNPs overlapping with the SGVP dataset were included in the analyses. As expected, the performances of SEEKIN and PC-Relate were highly similar, because genotypes are accurate at SNPs within deeply sequenced target regions (**Fig 6A and 6B**; **Table 3**). For simulated polygenic traits of h^2^ = 0.5 heritability, the targeted SNPs were able to capture ~86% of heritability using the kinship matrix from either SEEKIN or PC-Relate (estimated h^2^ = 0.43 after averaging across 1000 replicates, **Fig 7**). When we expanded our analyses to 1,054,229 SNPs across both target and off-target regions, the RMSE for SEEKIN estimates was reduced by ~50% across different relatedness types and the empirical bias remained close to 0 (**Fig 6C**). Using the improved kinship estimates, the estimated heritability was increased to 0.49, capturing ~98% of total heritability. In contrast, PC-Relate underestimated kinship coefficients by ~7% for closely related pairs after including off-target data (**Fig 6D**), leading to ~4% overestimation of the heritability. If we down sampled the off-target data to 0.15X, it became more evident that the heritability was overestimated by ~18% because PC-Relate underestimated kinship coefficients when analyzing inaccurate off-target genotypes (**Fig 7**). In comparison, the estimated heritability based on the kinship matrix from SEEKIN dropped from 0.49 to 0.46 (~8% underestimation) because less information was captured by 0.15X off-target data. We also tested if our noisy estimation of self-kinship coefficients affects heritability analysis. By replacing the diagonal elements in the estimated kinship matrices with ones or the values estimated from array genotyping data, our heritability estimates remained almost the same, suggesting the noises in our estimated self-kinship coefficients do not introduce bias in heritability analysis.

**Table 3:** Comparison of kinship estimation with and without off-target data in WES of 762 Chinese and Malays.

Dataset	Method	Unrelated (289,205 pairs)		3^rd^ degree (148 pairs)		2^nd^ degree (147pairs)		PO/FS (437 pairs)		Self-kinship (762 individuals)	
		RMSE	BIAS	RMSE	BIAS	RMSE	BIAS	RMSE	BIAS	RMSE	BIAS
Target	SEEKIN	0.006	-0.001	0.007	-0.001	0.008	-0.003	0.010	-0.004	0.018	-0.005
	PC-Relate	0.005	0.000	0.007	-0.001	0.008	-0.004	0.012	-0.008	0.022	-0.017
Target + off-target	SEEKIN	0.003	-0.001	0.003	0.001	0.004	0.001	0.007	0.003	0.017	0.002
	PC-Relate	0.002	0.000	0.004	-0.004	0.009	-0.009	0.019	-0.019	0.026	-0.021

Evaluation was based on SNPs overlapped with the SGVP dataset in the BEAGLE+1KG3 call set of 762 individuals for both SEEKIN and PC-Relate. 40,824 SNPs within target regions and 1,054,229 SNPs across target and off-target regions were included in the analyses. RMSE is the root mean squared error and BIAS is defined as the mean difference to the array-based estimates from PC-Relate for each type of relatedness. Negative values of BIAS suggest underestimation for results based on sparse sequencing data and vice versa.

Finally, we estimated heritability for 10 metabolic traits available in the Singapore Living Biobank dataset, adjusting for covariates of age, age^2^, sex, and two ancestry PCs (**Materials and Methods**). When we used the kinship matrix derived from array genotyping data, our heritability estimates were higher than the previously reported values based on unrelated samples but smaller than the values reported by twin studies (**Table 4**) [57–59]. Although heritability estimates are not directly comparable across studies due to differences in the pedigree structure and population background, the relative values for different traits in the same study are comparable. For example, we found cholesterol levels (HDL and LDL) to be more heritable than blood pressure measurements (DBP and SBP), which is consistent with previous studies [57–59]. For WES data, we used the kinship matrices derived from SEEKIN. As shown in **Table 4**, heritability estimates based on SNPs within target regions were consistently smaller than the values based on genome-wide array genotyping data by a minimum of 4% (for HbA1C) to a maximum of 29% (for DBP). After including off-target SNPs, WES-based estimates of heritability became much closer to the array-based estimates (from ≤1% difference for HbA1C, DBP, and TC to a maximum of 6% difference for FBG). These results, together with the simulations, suggest that our SEEKIN method is useful for WES studies to improve kinship estimation and downstream analyses such as estimation of trait heritability, by properly incorporating sparse data from off-target regions.

**Table 4:** Heritability estimation for 10 metabolic traits in 762 related Chinese and Malays.

Trait	Sample size	OmniExpress array (435,314 SNPs)	WES target + off-target (1,054,229 SNPs)	WES target only (40,824 SNPs)
BMI	762	0.587 (0.091)*	0.554 (0.090)	0.553 (0.087)
WHR	762	0.355 (0.096)	0.343 (0.092)	0.319 (0.087)
SBP	752	0.172 (0.098)	0.164 (0.098)	0.157 (0.090)
DBP	734	0.262 (0.099)	0.265 (0.097)	0.187 (0.089)
TC	761	0.523 (0.086)	0.517 (0.086)	0.438 (0.083)
LDL	761	0.602 (0.087)	0.593 (0.084)	0.470 (0.086)
HDL	761	0.658 (0.077)	0.632 (0.077)	0.576 (0.079)
TG	628	0.609 (0.101)	0.588 (0.010)	0.534 (0.099)
FBG	628	0.402 (0.105)	0.378 (0.103)	0.338 (0.101)
HbA1C	683	0.572 (0.092)	0.570 (0.090)	0.549 (0.089)

The pairwise relatedness matrix (2**Φ**) was estimated by PC-Relate for array genotyping data and by SEEKIN for sequencing data, based on common SNPs overlapped with the SGVP dataset. Trait heritability was estimated using a linear mixed model, adjusting for age, age^2^, sex, and the first two ancestry PCs.

Abbreviations of traits: BMI, body-mass index; WHR, waist-to-hip ratio; SBP, systolic blood pressure; DBP, diastolic blood pressure; TC, total cholesterol; LDL, low-density lipoprotein; HDL, high-density lipoprotein; TG, triglycerides; FBG,  fasting blood glucose; HbA1C, hemoglobin A1C.

* Values in the parenthesis indicate standard errors of the heritability estimates.

### Computational efficiency of SEEKIN

The whole SEEKIN analysis pipeline involved several steps starting from BAM files, including (1) genotype calling using BEAGLE, (2) ancestry estimation using LASER, (3) individual-specific allele frequency estimation using SEEKIN, and (4) kinship estimation using SEEKIN. For homogeneous samples, steps (2) and (3) can be skipped. As an example, we recorded the computational time of each step in the analysis of the BEAGLE+1KG3 call set for 762 individuals at ~0.15X. The BEAGLE step cost ~680 CPU days and ~1.7 wall-clock days when we split each chromosome into small chunks and ran the analysis in massive parallelization with 400 CPUs. We note that although the BEAGLE step is computationally intensive, especially with a large reference panel, it is a necessary step for all methods in analyzing shallow sequencing data. The LASER step cost ~34 CPU hours to place 762 individuals onto the ancestry map generated by the SGVP panel. The LASER step is scalable to large datasets because the computational time of LASER scales linearly to the study sample size and the analysis can be easily parallelized [38,39]. The last two steps using SEEKIN were fast; estimation of individual-specific allele frequencies across 1,285,277 SGVP SNPs cost only ~18 CPU minutes, and estimation of kinship coefficients based on the SEEKIN-het estimator cost ~116 CPU minutes.

In application to high-quality genotyping data, we do not need to process raw sequencing data so that the computationally intensive BEAGLE step can be skipped and the LASER step can run with a much faster algorithm for genotyping data [39]. To test the applicability of SEEKIN in large genotyping datasets, we further benchmarked the performance of kinship estimation using SEEKIN and existing methods based on two synthetic datasets of N = 10,000 individuals, generated by sampling with replacement from the Singapore Living Biobank sample. One dataset consists of M = 100,000 SNPs (100K dataset) and the other consists of M = 1,000,000 SNPs (1M dataset). For all evaluations, we set the number of CPUs to 10 if the software program supports multi-threading. As shown in **Table 5**, SEEKIN is both fast and memory efficient. The computational time of SEEKIN scales linearly to the number of SNPs and the memory usage remains constant (2.8 GB for SEEKIN-hom and 3.8 GB for SEEKIN-het). The higher memory cost for SEEKIN-het is due to the storage of individual-specific allele frequencies. The likelihood method, RelateAdmix, is computationally intensive and could not finish within 100 hours even for the smaller 100K dataset. In contrast, the moment methods are fast. SEEKIN-hom used 13 minutes to analyze the 100K dataset, while GCTA and KING only spent ~3 minutes. In the heterogeneous setting, SEEKIN-het spent 55 minutes, about 20 times faster than REAP and 45 times faster than PC-Relate. For the 1M dataset, only KING, SEEKIN-hom and SEEKIN-het managed to complete within 100 hours given 50 GB memory. Therefore, in addition to its unique capability for analyzing sparse sequencing data, SEEKIN is also useful for analyzing high-quality genotype data due to its computational efficiency and scalability to large datasets.

**Table 5:** Computational costs for kinship estimation software programs.

Estimator type	Method	Version	No. of CPUs	M = 100,000 SNPs		M = 1,000,000 SNPs	
				Wall-clock time	Peak memory	Wall-clock time	Peak memory
For homogeneous samples	SEEKIN-hom	v1.0	10	13 mins	2.8 GB	116 mins	2.8 GB
	KING	v2.09	10	3.0 mins	0.6 GB	30.3 mins	4.8 GB
	GCTA	v1.25.3	10	3.3 mins	5.8 GB	-	>50 GB
For heterogeneous samples with population structure and admixture	SEEKIN-het	v1.0	10	55 mins	3.8 GB	662 mins	3.8 GB
	REAP	v1.2	10	1168 mins	3.5 GB	>100 hours	-
	PC-Relate	v2.1.6	1	2550 mins	15.0 GB	>100 hours	-
	RelateAdmix	v0.14	1	>100 hours	-	>100 hours	-

Evaluations were based on two synthetic datasets of 10,000 individuals sampled with replacement from the WES data of 762 Chinese and Malays. We set the number of CPUs to 10 if the software program supports multi-threading feature. For all methods, we only evaluated computational cost for kinship estimation, excluding data preparation steps such as genotype calling and calculation of individual allele frequencies. For SEEKIN, we processed SNPs in blocks of size L = 10,000. PC-Relate was implemented in the R package “GENESIS” and the version number is for the “GENESIS” package. Tests were run on a high-performance computing cluster with Intel Xeon CPUs (2.8 GHz). Jobs were terminated if the memory usage exceeded 50 gigabytes (GB) or the run time exceeded 100 hours

## Discussion

In this study, we have developed moment estimators to infer kinship coefficients using sparse sequencing data for both homogeneous samples and heterogeneous samples with population structure and admixture. We have implemented our method into a computationally efficient and scalable software program named SEEKIN. Under certain model assumptions, our SEEKIN estimators share the same expectations as existing consistent estimators developed for high-quality genotyping data (GCTA [5] and PC-Relate [21]). Based on extensive evaluation on empirical datasets, we have demonstrated that SEEKIN can accurately estimate kinship coefficients using sparse sequencing data at ~0.15X, which corresponds to the typical off-target depth in target sequencing experiments. Existing methods, without accounting for the genotype uncertainty, substantially underestimate kinship coefficients when applied to sparse sequencing data. Such patterns persist even when the sequencing depth increases to ~0.75X. For WES studies, SEEKIN can improve kinship estimation by properly incorporating off-target sequencing data, as compared to the conventional analysis solely based on genotypes from deeply sequenced exonic regions.

Off-target reads, as byproducts of target sequencing experiments, are sparsely distributed genome-wide. The total amount of off-target reads, however, is of the same magnitude as the number of reads aligned to the target regions. Rather than discarding the vast amount of off-target data, we previously proposed to use off-target data to infer individual ancestry and control for population structure using our LASER method [38,39]. Now with the SEEKIN method, we can also control for family relatedness in target sequencing studies without additional genotyping data. Such an advancement is important because population structure and family relatedness are major confounders in genetic association studies and unexpected cryptic relatedness is prevalent in many datasets [60]. Because the kinship matrix is often used to model phenotype correlation in mixed models, our method also enables a variety of downstream analyses for target sequencing studies, including estimation of trait heritability and imputation of missing phenotypes [7,8]. In addition to target sequencing experiments, sparse human sequencing data can be extracted from metagenomic sequencing data across different human body sites [61]. We envision that both SEEKIN and LASER can be potentially used to infer the genetic background of human hosts, which might help explain patterns in microbiome composition across different individuals [61].

Our method leverages the LD information shared among study individuals and an external reference panel, such as the 1KG3 dataset, to analyze low-coverage sequencing data. Similar ideas of using LD between neighboring genetic markers have recently been proposed for matching forensic samples, which is a special case of identifying monozygotic twins in the inference of genetic relatedness, using either low-coverage sequencing data [29] or disjoint marker sets [62]. When an external reference panel is not available, LD information can be learnt from study individuals alone, especially when the sample size is large. Such LD-based imputation approaches not only increase the number of SNPs shared by any pair of individuals but also improve the overall genotyping accuracy [26,41].

We have shown that SEEKIN performs much better on the BEAGLE+1KG3 call sets than the BEAGLE call sets without a reference panel. As more human genomes are sequenced, we expect to achieve better performance in analyzing sparse sequencing data by utilizing larger and more relevant reference panels. Such improvement has been demonstrated for genotype imputation, where imputation accuracy increases as the size of the reference panel increases [63]. Large reference panels, however, are often not available for studies of non-human species, including many molecular ecology studies of wild animals based on non-invasive DNA samples, where inference of kinship from shallow sequencing data is of interests [30]. For these studies, the strategy of phasing without reference will be useful, and the performance of SEEKIN is expected to improve as the study sample size and sequencing depth increase.

We account for the genotype uncertainty using the statistical model proposed by Hu *et al*. [46]. The model (Eq 1) expresses the expectation of imputed dosage as a weighted sum of the true genotype and the mean genotype of the reference panel, with the weight given by the estimated dosage r^2^. For Eq (1) to hold, we need well calibrated genotype probabilities so that the imputed dosage and the estimated r^2^ reflect the genuine genotype uncertainty [46]. We examined the genotype probabilities output by BEAGLE in our examples by comparing to the array data (**S4 Fig**). We found that at ~0.75X depth, the genotype probabilities were well calibrated for both phasing with and without a reference panel. As the sequencing depth dropped to ~0.15X, the calibration remains good when phasing with the 1KG3 reference panel, but becomes inaccurate when phasing without reference panel. These results might explain why SEEKIN slightly underestimates kinship coefficients for the BEAGLE call sets at 0.15X (**Figs 3D** and **5A**). Even though we have modeled genotype uncertainty using dosage r^2^ in our estimators, we excluded SNPs with low quality (r^2^<0.5) for two reasons. First, Hu *et al*. [46] have shown that Eq (1) might not hold when r^2^ is close to 0. Second, low-quality SNPs contain less information and more noise, and thus might reduce the estimation accuracy when the quality fall below a certain threshold. We tested a lower threshold by including SNPs with r^2^>0.3, and found that SEEKIN produced similar results in comparison to using SNPs with r^2^>0.5, while the downward bias observed in the other methods became more evident (**S5 Fig** and **S6 Fig**).

Another assumption we made in the derivation of SEEKIN estimators is that residuals of Eq (1) are independent for different individuals (**S1 Text**). This is a reasonable assumption for sparse sequencing data because the variation in the residuals of imputed dosage is dominated by the randomness in the genomic distribution of sequence reads, which are independent for different sequenced samples. Nevertheless, this assumption does not strictly hold because we expect correlated residuals for related individuals due to their correlated genotypes. We cannot make this assumption for imputed array genotyping data because the input genotypes are highly correlated for closely related individuals. In an extreme example of monozygotic twins, the input array genotypes are identical and thus the imputed dosages are also identical, even though imputation might be inaccurate. For this reason, when applied to the imputed GWAS data, the underestimation for existing methods is largely reduced in comparison to the low-coverage sequencing setting, while SEEKIN overestimates kinship coefficients. Overall, SEEKIN performs well in the low-coverage sequencing datasets we have tested, suggesting that SEEKIN is robust to moderate violation of the assumptions, including independent residuals in the Eq (1) and accurate calibration of genotype probabilities.

Finally, our model implicitly assumes that the level of genotype uncertainty is similar among study individuals, which is reflected by the estimated dosage r^2^ for each SNP. This assumption posts a potential limitation on SEEKIN that it is not suitable to estimate kinship coefficients between two batches of samples with dramatic quality differences. For example, we cannot apply SEEKIN to identify cryptic relatedness between individuals from a WES dataset with ~1X off-target reads and individuals from a target sequencing dataset with ~0.2X off-target reads. For future work, we can generalize our kinship estimators to such scenarios by allowing for two r^2^ values, one for each dataset, to model different levels of genotype uncertainty in the datasets. A more general approach is to directly use genotype probabilities from each individual, instead of relying on a single estimated r^2^ statistic, to model genotype uncertainty. With these extensions, we can also identify potential relatedness between sequenced samples and array genotyped samples by treating the array genotyping data as accurate (i.e., r^2^ = 1 or genotype probability equal to 1). The ability to infer relatedness across different studies will be useful to help select samples to include in joint association analyses or in further biological experiments.

## Supporting information

S1 Text Expectations and variances of the SEEKIN estimators. (PDF)

S1 Table Performance of relationship classification based on homogeneous kinship estimators in ~0.15X sequencing data of 254 Chinese. (DOCX)

S2 Table Performance of homogeneous kinship estimators in ~0.75X sequencing data of 254 Chinese. (DOCX)

S3 Table Performance of relationship classification based on homogeneous kinship estimators in ~0.75X sequencing data of 254 Chinese. (DOCX)

S4 Table Performance of heterogeneous kinship estimators in ~0.75X sequencing data of 762 Chinese and Malays. (DOCX)

S5 Table Performance of relationship classification based on heterogeneous kinship estimators in ~0.15X sequencing data of 762 Chinese and Malays. (DOCX)

S6 Table Performance of relationship classification based on heterogeneous kinship estimators in ~0.75X sequencing data of 762 Chinese and Malays. (DOCX)

S1 Fig Illustration of the “single producer/consumer” design in the SEEKIN software. A single-threading “producer” job scans the input files, extracts required information for each SNP, and packs into a data block for every *L* SNPs. These data blocks are stored in the buffer, labeled as the blocking queue. Concurrently, a “consumer” job takes the data blocks one by one, performs multi-threading computation, and returns results. The results from different blocks are automatically combined after all blocks are analyzed. The “producer” and the “consumer” are synchronized through the blocking queue; the “producer” will become inactive if the blocking queue is full, and the “consumer” will become inactive if the blocking queue is empty. The best performance is achieved when production and consumption are balanced (i.e., the blocking queue is neither full nor empty).
(TIF)

S2 Fig Performance of homogeneous kinship estimators in ~0.75X sequencing data of 254 Chinese. In each panel, we compared sequence-based estimates (*ϕ*_seq_, y-axis) with the array-based estimates from PC-Relate (*ϕ*_array_, x-axis). Colored circles represent kinship coefficients between two individuals and different types of relatedness were determined in Fig 2. Grey crosses represent self-kinship coefficients. We evaluated lcMLkin (A), GCTA (B, E, H), KING (C, F, I), and SEEKIN (D, G) using the bcftools call set (A-C), the BEAGLE call set (D-F), and the BEAGLE+1KG3 call set (G-I). Note that KING does not estimate self-kinship coefficients.
(TIF)

S3 Fig Performance of heterogeneous kinship estimators in ~0.75X sequencing data of 762 Chinese and Malays. In each panel, we compared sequence-based estimates (*ϕ*_seq_, y-axis) with the array-based estimates from PC-Relate (*ϕ*_array_, x-axis). Colored circles represent kinship coefficients between two individuals and different types of relatedness were determined in Fig 2. Grey crosses represent self-kinship coefficients. We evaluated SEEKIN (A, E), PC-Relate (B, F), REAP (C, G), and RelateAdmix (D, H) using the BEAGLE call set (A-D), and the BEAGLE+1KG3 call set (E-H). We only included SNPs overlapping with the SGVP dataset in the analyses, because we used the SGVP dataset as the reference panel to estimate individual-specific allele frequencies for SEEKIN, REAP and RelateAdmix.
(TIF)

S4 Fig Calibration of posterior genotype probabilities from BEAGLE for different sequencing datasets. For each dataset, we binned the genotype probabilities into 100 bins spaced by 0.01 from 0 to 1 (x-axis). For each bin, we calculated the proportion of correct genotypes by comparing to the array genotypes (y-axis). The number of genotypes in each bin is color-coded according to the logarithmic scale in the color bar. When the genotype probabilities are well calibrated, we expect all data points on the diagonal. (A) BEAGLE call set for 254 Chinese at 0.15X. (B) BEAGLE+1KG3 call set for 254 Chinese at 0.15X. (C) BEAGLE call set for 762 Chinese and Malays at 0.15X. (D) BEAGLE+1KG3 call set for 762 Chinese and Malays at 0.15X. (E) BEAGLE call set for 254 Chinese at 0.75X. (F) BEAGLE+1KG3 call set for 254 Chinese at 0.75X. (G) BEAGLE call set for 762 Chinese and Malays at 0.75X. (H) BEAGLE+1KG3 call set for 762 Chinese and Malays at 0.75X.
(TIF)

S5 Fig Performance of homogeneous kinship estimators in ~0.15X sequencing data of 254 Chinese using SNPs with r^2^>0.3. In each panel, we compared sequence-based estimates (*ϕ*_seq_, y-axis) with the array-based estimates from PC-Relate (*ϕ*_array_, x-axis). Colored circles represent kinship coefficients between two individuals and different types of relatedness were determined in Fig 2. Grey crosses represent self-kinship coefficients. We evaluated SEEKIN (A, D), GCTA (B, E), and KING (C, F) using the BEAGLE call set (A-C), and the BEAGLE+1KG3 call set (D-F). Note that KING does not estimate self-kinship coefficients.
(TIF)

S6 Fig Performance of heterogeneous kinship estimators in ~0.75X sequencing data of 762 Chinese and Malays using SNPs with r^2^>0.3. In each panel, we compared sequence-based estimates (*ϕ*_seq_, y-axis) with the array-based estimates from PC-Relate (*ϕ*_array_, x-axis). Colored circles represent kinship coefficients between two individuals and different types of relatedness were determined in Fig 2. Grey crosses represent self-kinship coefficients. We evaluated SEEKIN (A, E), PC-Relate (B, F), REAP (C, G), and RelateAdmix (D, H) using the BEAGLE call set (A-D), and the BEAGLE+1KG3 call set (E-H). We only included SNPs overlapping with the SGVP dataset in the analyses, because we used the SGVP dataset as the reference panel to estimate individual-specific allele frequencies for SEEKIN, REAP and RelateAdmix.
(TIF)

## References

[1] Kang HM, Sul JH, Service SK, Zaitlen NA, Kong SY, et al (2010) Variance component model to account for sample structure in genome-wide association studies. Nat Genet 42: 348–354. doi: 10.1038/ng.548 20208533 10.1038/ng.548 PMC3092069

[2] Zhou X, Stephens M (2012) Genome-wide efficient mixed-model analysis for association studies. Nat Genet 44: 821–824. doi: 10.1038/ng.2310 22706312 10.1038/ng.2310 PMC3386377

[3] Lippert C, Listgarten J, Liu Y, Kadie CM, Davidson RI, et al (2011) FaST linear mixed models for genome-wide association studies. Nat Methods 8: 833–835. doi: 10.1038/nmeth.1681 21892150 10.1038/nmeth.1681

[4] Chen H, Wang C, Conomos MP, Stilp AM, Li Z, et al (2016) Control for Population Structure and Relatedness for Binary Traits in Genetic Association Studies via Logistic Mixed Models. Am J Hum Genet 98: 653–666. doi: 10.1016/j.ajhg.2016.02.012 27018471 10.1016/j.ajhg.2016.02.012 PMC4833218

[5] Yang J, Benyamin B, McEvoy BP, Gordon S, Henders AK, et al (2010) Common SNPs explain a large proportion of the heritability for human height. Nat Genet 42: 565–569. doi: 10.1038/ng.608 20562875 10.1038/ng.608 PMC3232052

[6] Tenesa A, Haley CS (2013) The heritability of human disease: estimation, uses and abuses. Nat Rev Genet 14: 139–149. doi: 10.1038/nrg3377 23329114 10.1038/nrg3377

[7] Dahl A, Iotchkova V, Baud A, Johansson A, Gyllensten U, et al (2016) A multiple-phenotype imputation method for genetic studies. Nat Genet 48: 466–472. doi: 10.1038/ng.3513 26901065 10.1038/ng.3513 PMC4817234

[8] Zhou X, Carbonetto P, Stephens M (2013) Polygenic modeling with bayesian sparse linear mixed models. PLoS Genet 9: e1003264 doi: 10.1371/journal.pgen.1003264 23408905 10.1371/journal.pgen.1003264 PMC3567190

[9] Weir BS, Anderson AD, Hepler AB (2006) Genetic relatedness analysis: modern data and new challenges. Nat Rev Genet 7: 771–780. doi: 10.1038/nrg1960 16983373 10.1038/nrg1960

[10] Thompson EA (1975) The estimation of pairwise relationships. Ann Hum Genet 39: 173–188. 1052764 10.1111/j.1469-1809.1975.tb00120.x

[11] Speed D, Balding DJ (2015) Relatedness in the post-genomic era: is it still useful? Nat Rev Genet 16: 33–44. doi: 10.1038/nrg3821 25404112 10.1038/nrg3821

[12] Milligan BG (2003) Maximum-likelihood estimation of relatedness. Genetics 163: 1153–1167. 12663552 10.1093/genetics/163.3.1153 PMC1462494

[13] Anderson AD, Weir BS (2007) A maximum-likelihood method for the estimation of pairwise relatedness in structured populations. Genetics 176: 421–440. doi: 10.1534/genetics.106.063149 17339212 10.1534/genetics.106.063149 PMC1893072

[14] Choi Y, Wijsman EM, Weir BS (2009) Case-control association testing in the presence of unknown relationships. Genet Epidemiol 33: 668–678. doi: 10.1002/gepi.20418 19333967 10.1002/gepi.20418 PMC2790016

[15] Queller DC, Goodnight KF (1989) Estimating relatedness using genetic markers. Evolution 43: 258–275. doi: 10.1111/j.1558-5646.1989.tb04226.x 28568555 10.1111/j.1558-5646.1989.tb04226.x

[16] Lynch M, Ritland K (1999) Estimation of pairwise relatedness with molecular markers. Genetics 152: 1753–1766. 10430599 10.1093/genetics/152.4.1753 PMC1460714

[17] Wang J (2002) An estimator for pairwise relatedness using molecular markers. Genetics 160: 1203–1215. 11901134 10.1093/genetics/160.3.1203 PMC1462003

[18] Manichaikul A, Mychaleckyj JC, Rich SS, Daly K, Sale M, et al (2010) Robust relationship inference in genome-wide association studies. Bioinformatics 26: 2867–2873. doi: 10.1093/bioinformatics/btq559 20926424 10.1093/bioinformatics/btq559 PMC3025716

[19] Wang J (2011) Unbiased relatedness estimation in structured populations. Genetics 187: 887–901. doi: 10.1534/genetics.110.124438 21212234 10.1534/genetics.110.124438 PMC3063680

[20] Thornton T, Tang H, Hoffmann TJ, Ochs-Balcom HM, Caan BJ, et al (2012) Estimating kinship in admixed populations. Am J Hum Genet 91: 122–138. doi: 10.1016/j.ajhg.2012.05.024 22748210 10.1016/j.ajhg.2012.05.024 PMC3397261

[21] Conomos MP, Reiner AP, Weir BS, Thornton TA (2016) Model-free Estimation of Recent Genetic Relatedness. Am J Hum Genet 98: 127–148. doi: 10.1016/j.ajhg.2015.11.022 26748516 10.1016/j.ajhg.2015.11.022 PMC4716688

[22] Moltke I, Albrechtsen A (2014) RelateAdmix: a software tool for estimating relatedness between admixed individuals. Bioinformatics 30: 1027–1028. doi: 10.1093/bioinformatics/btt652 24215025 10.1093/bioinformatics/btt652

[23] Alexander DH, Novembre J, Lange K (2009) Fast model-based estimation of ancestry in unrelated individuals. Genome Res 19: 1655–1664. doi: 10.1101/gr.094052.109 19648217 10.1101/gr.094052.109 PMC2752134

[24] Tang H, Peng J, Wang P, Risch NJ (2005) Estimation of individual admixture: analytical and study design considerations. Genet Epidemiol 28: 289–301. doi: 10.1002/gepi.20064 15712363 10.1002/gepi.20064

[25] Conomos MP, Miller MB, Thornton TA (2015) Robust inference of population structure for ancestry prediction and correction of stratification in the presence of relatedness. Genet Epidemiol 39: 276–293. doi: 10.1002/gepi.21896 25810074 10.1002/gepi.21896 PMC4836868

[26] Li Y, Sidore C, Kang HM, Boehnke M, Abecasis GR (2011) Low-coverage sequencing: implications for design of complex trait association studies. Genome Res 21: 940–951. doi: 10.1101/gr.117259.110 21460063 10.1101/gr.117259.110 PMC3106327

[27] The CONVERGE Consortium (2015) Sparse whole-genome sequencing identifies two loci for major depressive disorder. Nature 523: 588–591. doi: 10.1038/nature14659 26176920 10.1038/nature14659 PMC4522619

[28] The 1000 Genomes Project Consortium (2015) A global reference for human genetic variation. Nature 526: 68–74. doi: 10.1038/nature15393 26432245 10.1038/nature15393 PMC4750478

[29] Vohr SH, Buen Abad Najar CF, Shapiro B, Green RE (2015) A method for positive forensic identification of samples from extremely low-coverage sequence data. BMC Genomics 16: 1034 doi: 10.1186/s12864-015-2241-6 26643904 10.1186/s12864-015-2241-6 PMC4672566

[30] Snyder-Mackler N, Majoros WH, Yuan ML, Shaver AO, Gordon JB, et al (2016) Efficient Genome-Wide Sequencing and Low-Coverage Pedigree Analysis from Noninvasively Collected Samples. Genetics 203: 699–714. doi: 10.1534/genetics.116.187492 27098910 10.1534/genetics.116.187492 PMC4896188

[31] Martin MD, Jay F, Castellano S, Slatkin M (2017) Determination of genetic relatedness from low-coverage human genome sequences using pedigree simulations. Mol Ecol. 10.1111/mec.14188 PMC6485253 28543951

[32] Bamshad MJ, Ng SB, Bigham AW, Tabor HK, Emond MJ, et al (2011) Exome sequencing as a tool for Mendelian disease gene discovery. Nat Rev Genet 12: 745–755. doi: 10.1038/nrg3031 21946919 10.1038/nrg3031

[33] Zhan X, Larson DE, Wang C, Koboldt DC, Sergeev YV, et al (2013) Identification of a rare coding variant in complement 3 associated with age-related macular degeneration. Nat Genet 45: 1375–1379. doi: 10.1038/ng.2758 24036949 10.1038/ng.2758 PMC3812337

[34] Nejentsev S, Walker N, Riches D, Egholm M, Todd JA (2009) Rare variants of IFIH1, a gene implicated in antiviral responses, protect against type 1 diabetes. Science 324: 387–389. doi: 10.1126/science.1167728 19264985 10.1126/science.1167728 PMC2707798

[35] Rivas MA, Beaudoin M, Gardet A, Stevens C, Sharma Y, et al (2011) Deep resequencing of GWAS loci identifies independent rare variants associated with inflammatory bowel disease. Nat Genet 43: 1066–1073. doi: 10.1038/ng.952 21983784 10.1038/ng.952 PMC3378381

[36] Lek M, Karczewski KJ, Minikel EV, Samocha KE, Banks E, et al (2016) Analysis of protein-coding genetic variation in 60,706 humans. Nature 536: 285–291. doi: 10.1038/nature19057 27535533 10.1038/nature19057 PMC5018207

[37] Stessman HA, Xiong B, Coe BP, Wang T, Hoekzema K, et al (2017) Targeted sequencing identifies 91 neurodevelopmental-disorder risk genes with autism and developmental-disability biases. Nat Genet 49: 515–526. doi: 10.1038/ng.3792 28191889 10.1038/ng.3792 PMC5374041

[38] Wang C, Zhan X, Bragg-Gresham J, Kang HM, Stambolian D, et al (2014) Ancestry estimation and control of population stratification for sequence-based association studies. Nat Genet 46: 409–415. doi: 10.1038/ng.2924 24633160 10.1038/ng.2924 PMC4084909

[39] Wang C, Zhan X, Liang L, Abecasis GR, Lin X (2015) Improved ancestry estimation for both genotyping and sequencing data using projection Procrustes analysis and genotype imputation. Am J Hum Genet 96: 926–937. doi: 10.1016/j.ajhg.2015.04.018 26027497 10.1016/j.ajhg.2015.04.018 PMC4457959

[40] Lipatov M, Sanjeev K, Patro R, Veeramah K (2015) Maximum likelihood estimation of biological relatedness from low coverage sequencing data. bioRxiv: 023374.

[41] Pasaniuc B, Rohland N, McLaren PJ, Garimella K, Zaitlen N, et al (2012) Extremely low-coverage sequencing and imputation increases power for genome-wide association studies. Nat Genet 44: 631–635. doi: 10.1038/ng.2283 22610117 10.1038/ng.2283 PMC3400344

[42] Browning BL, Browning SR (2009) A unified approach to genotype imputation and haplotype-phase inference for large data sets of trios and unrelated individuals. Am J Hum Genet 84: 210–223. doi: 10.1016/j.ajhg.2009.01.005 19200528 10.1016/j.ajhg.2009.01.005 PMC2668004

[43] Li Y, Willer CJ, Ding J, Scheet P, Abecasis GR (2010) MaCH: using sequence and genotype data to estimate haplotypes and unobserved genotypes. Genet Epidemiol 34: 816–834. doi: 10.1002/gepi.20533 21058334 10.1002/gepi.20533 PMC3175618

[44] Howie B, Fuchsberger C, Stephens M, Marchini J, Abecasis GR (2012) Fast and accurate genotype imputation in genome-wide association studies through pre-phasing. Nat Genet 44: 955–959. doi: 10.1038/ng.2354 22820512 10.1038/ng.2354 PMC3696580

[45] Browning BL, Browning SR (2016) Genotype Imputation with Millions of Reference Samples. Am J Hum Genet 98: 116–126. doi: 10.1016/j.ajhg.2015.11.020 26748515 10.1016/j.ajhg.2015.11.020 PMC4716681

[46] Hu YJ, Li Y, Auer PL, Lin DY (2015) Integrative analysis of sequencing and array genotype data for discovering disease associations with rare mutations. Proc Natl Acad Sci U S A 112: 1019–1024. doi: 10.1073/pnas.1406143112 25583502 10.1073/pnas.1406143112 PMC4313847

[47] Li H, Handsaker B, Wysoker A, Fennell T, Ruan J, et al (2009) The Sequence Alignment/Map format and SAMtools. Bioinformatics 25: 2078–2079. doi: 10.1093/bioinformatics/btp352 19505943 10.1093/bioinformatics/btp352 PMC2723002

[48] Li H (2011) A statistical framework for SNP calling, mutation discovery, association mapping and population genetical parameter estimation from sequencing data. Bioinformatics 27: 2987–2993. doi: 10.1093/bioinformatics/btr509 21903627 10.1093/bioinformatics/btr509 PMC3198575

[49] Bhatia G, Patterson N, Sankararaman S, Price AL (2013) Estimating and interpreting F_ST_: The impact of rare variants. Genome Res 23: 1514–1521. doi: 10.1101/gr.154831.113 23861382 10.1101/gr.154831.113 PMC3759727

[50] Sanderson C, Curtin R (2016) Armadillo: a template-based C++ library for linear algebra. Journal of Open Source Software 1: 26–32.

[51] Win AM, Yen LW, Tan KH, Lim RB, Chia KS, et al (2015) Patterns of physical activity and sedentary behavior in a representative sample of a multi-ethnic South-East Asian population: a cross-sectional study. BMC Public Health 15: 318 doi: 10.1186/s12889-015-1668-7 25884916 10.1186/s12889-015-1668-7 PMC4391474

[52] Li H, Durbin R (2010) Fast and accurate long-read alignment with Burrows-Wheeler transform. Bioinformatics 26: 589–595. doi: 10.1093/bioinformatics/btp698 20080505 10.1093/bioinformatics/btp698 PMC2828108

[53] DePristo MA, Banks E, Poplin R, Garimella KV, Maguire JR, et al (2011) A framework for variation discovery and genotyping using next-generation DNA sequencing data. Nat Genet 43: 491–498. doi: 10.1038/ng.806 21478889 10.1038/ng.806 PMC3083463

[54] Teo YY, Sim X, Ong RT, Tan AK, Chen J, et al (2009) Singapore Genome Variation Project: a haplotype map of three Southeast Asian populations. Genome Res 19: 2154–2162. doi: 10.1101/gr.095000.109 19700652 10.1101/gr.095000.109 PMC2775604

[55] Kopelman NM, Mayzel J, Jakobsson M, Rosenberg NA, Mayrose I (2015) Clumpak: a program for identifying clustering modes and packaging population structure inferences across K. Mol Ecol Resour 15: 1179–1191. doi: 10.1111/1755-0998.12387 25684545 10.1111/1755-0998.12387 PMC4534335

[56] Wang C, Szpiech ZA, Degnan JH, Jakobsson M, Pemberton TJ, et al (2010) Comparing spatial maps of human population-genetic variation using Procrustes analysis. Stat Appl Genet Mol Biol 9: Article 13. 10.2202/1544-6115.1493 PMC2861313 20196748

[57] Browning SR, Browning BL (2013) Identity-by-descent-based heritability analysis in the Northern Finland Birth Cohort. Hum Genet 132: 129–138. doi: 10.1007/s00439-012-1230-y 23052944 10.1007/s00439-012-1230-y PMC3543768

[58] Wessel J, Moratorio G, Rao F, Mahata M, Zhang L, et al (2007) C-reactive protein, an 'intermediate phenotype' for inflammation: human twin studies reveal heritability, association with blood pressure and the metabolic syndrome, and the influence of common polymorphism at catecholaminergic/beta-adrenergic pathway loci. J Hypertens 25: 329–343. doi: 10.1097/HJH.0b013e328011753e 17211240 10.1097/HJH.0b013e328011753e

[59] Souren NY, Paulussen AD, Loos RJ, Gielen M, Beunen G, et al (2007) Anthropometry, carbohydrate and lipid metabolism in the East Flanders Prospective Twin Survey: heritabilities. Diabetologia 50: 2107–2116. doi: 10.1007/s00125-007-0784-z 17694296 10.1007/s00125-007-0784-z PMC2039867

[60] Pemberton TJ, Wang C, Li JZ, Rosenberg NA (2010) Inference of unexpected genetic relatedness among individuals in HapMap Phase III. Am J Hum Genet 87: 457–464. doi: 10.1016/j.ajhg.2010.08.014 20869033 10.1016/j.ajhg.2010.08.014 PMC2948801

[61] Blekhman R, Goodrich JK, Huang K, Sun Q, Bukowski R, et al (2015) Host genetic variation impacts microbiome composition across human body sites. Genome Biol 16: 191 doi: 10.1186/s13059-015-0759-1 26374288 10.1186/s13059-015-0759-1 PMC4570153

[62] Edge MD, Algee-Hewitt BFB, Pemberton TJ, Li JZ, Rosenberg NA (2017) Linkage disequilibrium matches forensic genetic records to disjoint genomic marker sets. Proc Natl Acad Sci U S A. 10.1073/pnas.1619944114 PMC5465933 28507140

[63] McCarthy S, Das S, Kretzschmar W, Delaneau O, Wood AR, et al (2016) A reference panel of 64,976 haplotypes for genotype imputation. Nat Genet 48: 1279–1283. doi: 10.1038/ng.3643 27548312 10.1038/ng.3643 PMC5388176

