# Supplementary material for: Estimation of kinship coefficient in structured and admixed populations using sparse sequencing data
Source: PLoS Genet. 2017 Sep 29;13(9):e1007021. doi: 10.1371/journal.pgen.1007021 (PMC5636172; doi:10.1371/journal.pgen.1007021)
Supplement: S1 Text — (PDF) [file pgen.1007021.s001.pdf]

Estimation of Kinship Coefficient in Structured and Admixed Populations using Sparse Sequencing Data

Supplementary Material

Jinzhuang Dou[1,*], Baoluo Sun[1,*], Xueling Sim[2], Jason D Hughes[3], Dermot F Reilly[3], E Shyong Tai[2,4,5], Jianjun Liu[5,6], Chaolong Wang[1,4,§]

[1] Computational and Systems Biology, Genome Institute of Singapore, Singapore, Singapore
[2] Saw Swee Hock School of Public Health, National University of Singapore, Singapore, Singapore
[3] Genetics, Merck Sharp & Dohme Corp., Kenilworth, New Jersey, United States of America
[4] Duke-NUS Medical School, National University of Singapore, Singapore, Singapore
[5] Yong Loo Lin School of Medicine, National University of Singapore, Singapore, Singapore
[6] Human Genetics, Genome Institute of Singapore, Singapore, Singapore

[*] These authors contribute equally to this work.
[§] Correspondence: wangcl@gis.a-star.edu.sg

S1 Text. Expectations and variances of the SEEKIN estimators

A. Expectations of the SEEKIN estimators

Here we derive the expectations of our proposed kinship estimators at a locus for homogeneous samples (Equations 4 and 5) and for samples with population structure and admixture (Equations 9 and 10). We show that our estimators share the same expectations with those of genotype-based estimators, under the assumptions that the residuals of Equation 1 (defined below) are uncorrelated for any pair of individuals and that the allele frequencies are accurately estimated.

Let $\varepsilon_{im} = \tilde{G}_{im} - \mathrm{E}\left(\tilde{G}_{im} \mid G_{im}, \bar{G}_{Rm}\right)$ be the residue term for individual i at the mth locus. Then the proposed kinship estimator (4) has the expression

$$\begin{aligned}
2\tilde{\phi}_{ijm} &= \frac{\left(\tilde{G}_{im} - 2\tilde{p}_m\right)\left(\tilde{G}_{jm} - 2\tilde{p}_m\right)}{2\tilde{p}_m\left(1-\tilde{p}_m\right)\left(\widehat{r_m^2}\right)^2} = \frac{\left(\tilde{G}_{im} - 2\tilde{p}_m - \varepsilon_{im} + \varepsilon_{im}\right)\left(\tilde{G}_{jm} - 2\tilde{p}_m - \varepsilon_{jm} + \varepsilon_{jm}\right)}{2\tilde{p}_m\left(1-\tilde{p}_m\right)\left(\widehat{r_m^2}\right)^2} \\
&= \frac{\left(\tilde{G}_{im} - 2\tilde{p}_m - \varepsilon_{im}\right)\left(\tilde{G}_{jm} - 2\tilde{p}_m - \varepsilon_{jm}\right) + \varepsilon_{im}\left(\tilde{G}_{jm} - 2\tilde{p}_m - \varepsilon_{jm}\right) + \varepsilon_{jm}\left(\tilde{G}_{im} - 2\tilde{p}_m - \varepsilon_{im}\right) + \varepsilon_{im}\varepsilon_{jm}}{2\tilde{p}_m\left(1-\tilde{p}_m\right)\left(\widehat{r_m^2}\right)^2} \quad \text{(A.1)} \\
&= \frac{\left(G_{im} - 2p_m\right)\left(G_{jm} - 2p_m\right)}{2\tilde{p}_m\left(1-\tilde{p}_m\right)} + \frac{\widehat{r_m^2}\left(G_{jm} - 2p_m\right)\varepsilon_{im} + \widehat{r_m^2}\left(G_{im} - 2p_m\right)\varepsilon_{jm} + \varepsilon_{im}\varepsilon_{jm}}{2\tilde{p}_m\left(1-\tilde{p}_m\right)\left(\widehat{r_m^2}\right)^2}
\end{aligned}$$

The last equality in (A.1) holds because we can derive from Equations (1) and (2) that

$$\tilde{G}_{im} - 2\tilde{p}_m - \varepsilon_{im} = \mathrm{E}\left(\tilde{G}_{im} \mid G_{im}, \bar{G}_{Rm}\right) - 2\tilde{p}_m \approx \widehat{r_m^2}(G_{im} - 2p_m). \tag{A.2}$$

Because $\mathrm{E}(\varepsilon_{im}) = 0$, under the assumptions that $\varepsilon_{im} \perp G_{jm}$ and $\varepsilon_{im} \perp \varepsilon_{jm}$ for any $i \neq j$ and that $\tilde{p}_m \approx p_m$, we have

$$\mathrm{E}\left(\tilde{\phi}_{ijm}\right) \approx \mathrm{E}\left[\frac{(G_{im} - 2p_m)(G_{jm} - 2p_m)}{2p_m(1-p_m)}\right] = \mathrm{E}\left(\hat{\phi}_{ijm}\right), \tag{A.3}$$

where $\hat{\phi}_{ijm}$ is the genotype-based estimator given in Equation (3) [1].

Next, we consider the self-kinship coefficient $\tilde{\phi}_{iim}$ given by Equation (5):

$$\mathrm{E}\left(\tilde{\phi}_{iim}\right) = \frac{\mathrm{E}\left(\tilde{G}_{im} - 2\tilde{p}_m\right)^2}{4\tilde{p}_m(1-\tilde{p}_m)\widehat{r_m^2}} = \frac{\mathrm{Var}\left(\tilde{G}_{im}\right)}{4\tilde{p}_m(1-\tilde{p}_m)\widehat{r_m^2}} = \frac{\mathrm{Var}(G_{im})}{4\tilde{p}_m(1-\tilde{p}_m)}. \tag{A.4}$$

The last equality in (A.4) arises from the definition of $\widehat{r_m^2} = \mathrm{Var}\left(\tilde{G}_{im}\right) / \mathrm{Var}(G_{im})$ [2]. Under an inbreeding model with inbreeding coefficient f_i, the frequencies of genotypes 0, 1, and 2 are given by $(1-p_m)^2 + p_m(1-p_m)f_i$, $2p_m(1-p_m)(1-f_i)$, and $p_m^2 + p_m(1-p_m)f_i$, respectively [3]. The variance of genotypes can be written as $\mathrm{Var}(G_{im}) = 2p_m(1-p_m)(1+f_i)$. Consequently,

$$\mathrm{E}\left(\tilde{\phi}_{iim}\right) = \frac{2p_m(1-p_m)(1+f_i)}{4\tilde{p}_m(1-\tilde{p}_m)} \approx \frac{1+f_i}{2}. \tag{A.5}$$

For samples with population structure and admixture, the proposed kinship estimator (9) can be written as

$$\begin{aligned} 2\tilde{\phi}_{ijm} &= \frac{\left(\tilde{G}_{im} - 2\tilde{u}_{im}\right)\left(\tilde{G}_{jm} - 2\tilde{u}_{jm}\right)}{2\sqrt{\hat{p}_{im}(1-\hat{p}_{im})\hat{p}_{jm}(1-\hat{p}_{jm})}\left(\widehat{r_m^2}\right)^2} = \frac{\left(\tilde{G}_{im} - 2\tilde{p}_m - 2\widehat{r_m^2}(\hat{p}_{im} - \hat{p}_m)\right)\left(\tilde{G}_{jm} - 2\tilde{p}_m - 2\widehat{r_m^2}(\hat{p}_{jm} - \hat{p}_m)\right)}{2\sqrt{\hat{p}_{im}(1-\hat{p}_{im})\hat{p}_{jm}(1-\hat{p}_{jm})}\left(\widehat{r_m^2}\right)^2} \\ &= \frac{\left[(G_{im} - 2p_m) - 2(\hat{p}_{im} - \hat{p}_m) + \varepsilon_{im}\left(\widehat{r_m^2}\right)^{-1}\right]\left[(G_{jm} - 2p_m) - 2(\hat{p}_{jm} - \hat{p}_m) + \varepsilon_{jm}\left(\widehat{r_m^2}\right)^{-1}\right]}{2\sqrt{\hat{p}_{im}(1-\hat{p}_{im})\hat{p}_{jm}(1-\hat{p}_{jm})}}, \end{aligned} \tag{A.6}$$

where the last equality follows from (A.2). We assume accurate estimation of individual-specific allele frequencies $\hat{p}_{im} \approx p_{im}$ for $i = 1, 2, ..., N$. Further assuming $\varepsilon_{im} \perp G_{jm}$ and $\varepsilon_{im} \perp \varepsilon_{jm}$ for any $i \neq j$, we have

$$\mathrm{E}\left(\tilde{\phi}_{ijm}\right) \approx \mathrm{E}\left[\frac{(G_{im} - 2p_{im})(G_{jm} - 2p_{jm})}{4\sqrt{p_{im}(1-p_{im})p_{jm}(1-p_{jm})}}\right] = \mathrm{E}\left(\hat{\phi}_{ijm}\right), \tag{A.7}$$

where $\hat{\phi}_{ijm}$ is the genotype-based PC-Relate kinship estimator (Equation 8 with $i \neq j$) and is a consistent estimator of ϕ_{ij} [4].

Finally, we derive the expectation of the self-kinship coefficient estimator by Equation (10), which can be written as

$$
\begin{aligned}
2\tilde{\phi}_{iim} &= \frac{\left(\tilde{G}_{im} - 2\tilde{u}^*_{im}\right)^2}{2\hat{p}_{im}\left(1-\hat{p}_{im}\right)\widehat{r_m^2}} = \frac{\left[\tilde{G}_{im} - 2\tilde{p}_m - 2\left(\hat{p}_{im} - \hat{p}_m\right)\sqrt{\widehat{r_m^2}}\right]^2}{2\hat{p}_{im}\left(1-\hat{p}_{im}\right)\widehat{r_m^2}} \\
&= \frac{\left(\tilde{G}_{im} - 2\tilde{p}_m\right)^2 - 4\left(\hat{p}_{im} - \hat{p}_m\right)\left(\tilde{G}_{im} - 2\tilde{p}_m\right)\sqrt{\widehat{r_m^2}} + 4\left(\hat{p}_{im} - \hat{p}_m\right)^2\widehat{r_m^2}}{2\hat{p}_{im}\left(1-\hat{p}_{im}\right)\widehat{r_m^2}}.
\end{aligned}
\tag{A.8}
$$

Because $\mathrm{E}\left[\left(\tilde{G}_{im} - 2\tilde{p}_m\right)^2\right] = \widehat{r_m^2}\,\mathrm{E}\left[\left(G_{im} - 2p_m\right)^2\right]$ and $\mathrm{E}\left(\tilde{G}_{im} - 2\tilde{p}_m\right) = \sqrt{\widehat{r_m^2}}\,\mathrm{E}\left(G_{im} - 2p_m\right) = 0$, we can substitute into the expectation of (A.8) and get

$$
\begin{aligned}
\mathrm{E}(\tilde{\phi}_{iim}) &= \mathrm{E}\left[\frac{\widehat{r_m^2}\left(G_{im} - 2p_m\right)^2 - 4\left(\hat{p}_{im} - \hat{p}_m\right)\left(G_{im} - 2p_m\right)\widehat{r_m^2} + 4\left(\hat{p}_{im} - \hat{p}_m\right)^2\widehat{r_m^2}}{4\hat{p}_{im}\left(1-\hat{p}_{im}\right)\widehat{r_m^2}}\right] \\
&= \mathrm{E}\left[\frac{\left(G_{im} - 2p_m - 2\left(\hat{p}_{im} - \hat{p}_m\right)\right)^2}{4\hat{p}_{im}\left(1-\hat{p}_{im}\right)}\right] \approx \mathrm{E}\left[\frac{\left(G_{im} - 2\hat{p}_{im}\right)^2}{4\hat{p}_{im}\left(1-\hat{p}_{im}\right)}\right] = \mathrm{E}(\hat{\phi}_{iim})
\end{aligned}
\tag{A.9}
$$

where $\hat{\phi}_{iim}$ is the genotype-based self-kinship estimator in PC-Relate (Equation 8 with $i = j$) and is a consistent estimator of $(1+f_i)/2$ [4].

B. Variances of the SEEKIN estimators for unrelated pairs

In this section, we derive the variances of our proposed SEEKIN estimators at a single locus for unrelated pairs in homogeneous samples (Equation 4) and in samples with population structure and admixture (Equation 9). We use the results to justify our choice of weight function when combining kinship estimates across loci under the inverse-variance weighting scheme. We do not derive the variances of kinship estimates for related pairs because the derivation is complicated without assuming independence between individuals. In practice, most pairs in a dataset are unrelated, so it is natural to choose a weight function based on unrelated pairs.

We first derive the variance of our SEEKIN estimator for unrelated pairs in a homogeneous population (Equation 4). According to (A.1) with the assumption that $\tilde{p}_m = p_m$, we have

$$\begin{aligned}\operatorname{Var}\left(2\tilde{\phi}_{ijm}\right) &= \operatorname{Var}\left[\frac{(G_{im}-2p_m)(G_{jm}-2p_m)}{2p_m(1-p_m)}+\frac{(G_{jm}-2p_m)\varepsilon_{im}}{2p_m(1-p_m)\widehat{r_m^2}}+\frac{(G_{im}-2p_m)\varepsilon_{jm}}{2p_m(1-p_m)\widehat{r_m^2}}+\frac{\varepsilon_{im}\varepsilon_{jm}}{2p_m(1-p_m)\left(\widehat{r_m^2}\right)^2}\right] \\ &= \operatorname{Var}\left[\left(\frac{G_{im}-2p_m}{\sqrt{2p_m(1-p_m)}}+\frac{\varepsilon_{im}}{\sqrt{2p_m(1-p_m)}\widehat{r_m^2}}\right)\left(\frac{G_{jm}-2p_m}{\sqrt{2p_m(1-p_m)}}+\frac{\varepsilon_{jm}}{\sqrt{2p_m(1-p_m)}\widehat{r_m^2}}\right)\right] \\ &= \operatorname{Var}\left(\frac{G_{im}-2p_m}{\sqrt{2p_m(1-p_m)}}+\frac{\varepsilon_{im}}{\sqrt{2p_m(1-p_m)}\widehat{r_m^2}}\right)\operatorname{Var}\left(\frac{G_{jm}-2p_m}{\sqrt{2p_m(1-p_m)}}+\frac{\varepsilon_{jm}}{\sqrt{2p_m(1-p_m)}\widehat{r_m^2}}\right) \\ &= \left(\frac{\operatorname{Var}(G_{im})}{2p_m(1-p_m)}+\frac{\operatorname{Var}(\varepsilon_{im})}{2p_m(1-p_m)\left(\widehat{r_m^2}\right)^2}\right)\left(\frac{\operatorname{Var}(G_{jm})}{2p_m(1-p_m)}+\frac{\operatorname{Var}(\varepsilon_{jm})}{2p_m(1-p_m)\left(\widehat{r_m^2}\right)^2}\right),\end{aligned} \tag{B.1}$$

in which, the last equity holds because $G_{im} \perp \varepsilon_{im}$ under the linear model in Equation (1). The second last equity in (B.1) holds because $\mathrm{E}(G_{im}-2p_m)=\mathrm{E}(\varepsilon_{im})=0$ and $G_{jm} \perp G_{im}, \varepsilon_{im}$ when individuals *i* and *j* are unrelated.

According to (A.2) and because $G_{im} \perp \varepsilon_{im}$, we have

$$\operatorname{Var}\left(\tilde{G}_{im}-2\tilde{p}_m\right)=\left(\widehat{r_m^2}\right)^2\operatorname{Var}(G_{im}-2p_m)+\operatorname{Var}(\varepsilon_{im}). \tag{B.2}$$

Because $\widehat{r_m^2}=\operatorname{Var}\left(\tilde{G}_{im}\right)\big/\operatorname{Var}(G_{im})$, $\operatorname{Var}\left(\tilde{G}_{im}-2\tilde{p}_m\right)=\operatorname{Var}\left(\tilde{G}_{im}\right)$, and $\operatorname{Var}(G_{im}-2p_m)=\operatorname{Var}(G_{im})$, we have

$$\operatorname{Var}(\varepsilon_{im})=\widehat{r_m^2}\left(1-\widehat{r_m^2}\right)\operatorname{Var}(G_{im}). \tag{B.3}$$

Substituting (B.3) into (B.1), the variance of our SEEKIN estimate of kinship between unrelated individuals in a homogeneous population can be written as

$$\operatorname{Var}\left(2\tilde{\phi}_{ijm}\right)=\left(\frac{\operatorname{Var}(G_{im})}{2p_m(1-p_m)\widehat{r_m^2}}\right)\left(\frac{\operatorname{Var}(G_{jm})}{2p_m(1-p_m)\widehat{r_m^2}}\right)=(1+f_i)(1+f_j)\left(\widehat{r_m^2}\right)^{-2}, \tag{B.4}$$

in which, f_i and f_j are inbreeding coefficients for individuals *i* and *j*, respectively. Under Hardy-Weinberg Equilibrium (HWE), we have $f_i = f_j = 0$ and $\operatorname{Var}\left(2\tilde{\phi}_{ijm}\right)=\left(\widehat{r_m^2}\right)^{-2}$.

The derivation for the variance of our SEEKIN estimator for unrelated pairs in a sample with population structure and admixture (Equation 9) is analogous, except that we assume that individual-specific allele frequencies are accurately estimated: $\hat{p}_{im}=p_{im}$ for $i=1,2,...,N$, and that $\operatorname{Var}(G_{im})=\operatorname{Var}(G_{im}-2p_{im})=2p_{im}(1-p_{im})(1+f_i)$ under the inbreeding model. Briefly, according to (A.6) and (B.3), we can derive that

$$
\begin{aligned}
\mathrm{Var}\left(2\tilde{\phi}_{ijm}\right) &= \mathrm{Var}\left[\left(\frac{\left(G_{im}-2p_{im}\right)}{\sqrt{2p_{im}\left(1-p_{im}\right)}}+\frac{\varepsilon_{im}}{\widehat{r_m^2}\sqrt{2p_{im}\left(1-p_{im}\right)}}\right)\left(\frac{\left(G_{jm}-2p_{jm}\right)}{\sqrt{2p_{jm}\left(1-p_{jm}\right)}}+\frac{\varepsilon_{jm}}{\widehat{r_m^2}\sqrt{2p_{jm}\left(1-p_{jm}\right)}}\right)\right] \\
&= \mathrm{Var}\left(\frac{G_{im}-2p_{im}}{\sqrt{2p_{im}\left(1-p_{im}\right)}}+\frac{\varepsilon_{im}}{\widehat{r_m^2}\sqrt{2p_{im}\left(1-p_{im}\right)}}\right)\mathrm{Var}\left(\frac{G_{jm}-2p_{jm}}{\sqrt{2p_{jm}\left(1-p_{jm}\right)}}+\frac{\varepsilon_{jm}}{\widehat{r_m^2}\sqrt{2p_{jm}\left(1-p_{jm}\right)}}\right) \qquad \text{(B.5)} \\
&= \left(\frac{\mathrm{Var}\left(G_{im}\right)}{2p_{im}\left(1-p_{im}\right)}+\frac{\mathrm{Var}\left(\varepsilon_{im}\right)}{\left(\widehat{r_m^2}\right)^2 2p_{im}\left(1-p_{im}\right)}\right)\left(\frac{\mathrm{Var}\left(G_{jm}\right)}{2p_{jm}\left(1-p_{jm}\right)}+\frac{\mathrm{Var}\left(\varepsilon_{jm}\right)}{\left(\widehat{r_m^2}\right)^2 2p_{jm}\left(1-p_{jm}\right)}\right) \\
&= \left(\frac{\mathrm{Var}\left(G_{im}\right)}{2p_{im}\left(1-p_{im}\right)\widehat{r_m^2}}\right)\left(\frac{\mathrm{Var}\left(G_{jm}\right)}{2p_{jm}\left(1-p_{jm}\right)\widehat{r_m^2}}\right) = \left(1+f_i\right)\left(1+f_j\right)\left(\widehat{r_m^2}\right)^{-2}.
\end{aligned}
$$

Supplementary References

1. Yang J, Benyamin B, McEvoy BP, Gordon S, Henders AK, et al. (2010) Common SNPs explain a large proportion of the heritability for human height. Nat Genet 42: 565-569.
2. Hu YJ, Li Y, Auer PL, Lin DY (2015) Integrative analysis of sequencing and array genotype data for discovering disease associations with rare mutations. Proc Natl Acad Sci U S A 112: 1019-1024.
3. Holsinger KE, Weir BS (2009) Genetics in geographically structured populations: defining, estimating and interpreting F_{ST}. Nat Rev Genet 10: 639-650.
4. Thornton T, Tang H, Hoffmann TJ, Ochs-Balcom HM, Caan BJ, et al. (2012) Estimating kinship in admixed populations. Am J Hum Genet 91: 122-138.
