# Supplementary material for: Estimation of kinship coefficient in structured and admixed populations using sparse sequencing data
Source: PLoS Genet. 2017 Sep 29;13(9):e1007021. doi: 10.1371/journal.pgen.1007021 (PMC5636172; doi:10.1371/journal.pgen.1007021)
Supplement: S1 Table — (DOCX) [file pgen.1007021.s002.docx]

**S1 Table. Performance of relationship classification based on homogeneous kinship estimators in ~0.15X sequencing data of 254 Chinese.**

| **Call set** | **Method** | **3^rd^ degree** | | **2^nd^ degree** | | **PO/FS** | |
| --- | --- | --- | --- | --- | --- | --- | --- |
|  |  | **Precision** | **Sensitivity** | **Precision** | **Sensitivity** | **Precision** | **Sensitivity** |
| Bcftools | lcMLkin | 0.009 | 1.000* | 0.950* | 1.000* | 1.000* | 1.000* |
|  | GCTA | 0.027* | 0.045 | 0.000 | 0.000 | -- | 0.000 |
|  | KING | 0.001 | 0.864 | -- | 0.000 | -- | 0.000 |
| BEAGLE | SEEKIN | 0.941* | 0.727* | 0.946* | 0.972* | 1.000* | 0.986* |
|  | GCTA | 0.029 | 0.045 | 0.022 | 0.083 | 1.000* | 0.082 |
|  | KING | 0.056 | 0.091 | 0.020 | 0.083 | 1.000* | 0.014 |
| BEAGLE+1KG3 | SEEKIN | 1.000* | 1.000* | 1.000* | 1.000* | 1.000* | 1.000* |
|  | GCTA | 0.800 | 0.727 | 0.800 | 0.889 | 1.000* | 0.945 |
|  | KING | 0.824 | 0.636 | 0.846 | 0.917 | 1.000* | 0.959 |

Precision is defined as the proportion of correct classification among all pairs of a relationship type inferred from the sequence-based kinship estimates. Sensitivity is defined as the proportion of correct classification among pairs of a relationship type inferred from the gold standard kinship estimates.

^*^ Highest values of precision or sensitivity in each call set and each relationship type.
