# Supplementary material for: Estimation of kinship coefficient in structured and admixed populations using sparse sequencing data
Source: PLoS Genet. 2017 Sep 29;13(9):e1007021. doi: 10.1371/journal.pgen.1007021 (PMC5636172; doi:10.1371/journal.pgen.1007021)
Supplement: S2 Table — (DOCX) [file pgen.1007021.s003.docx]

**S2 Table. Performance of homogeneous kinship estimators in ~0.75X sequencing data of 254 Chinese.**

| **Call set** | **Method** | **Unrelated**  **( 31,925 pairs)** | | **3^rd^ degree**  **( 22 pairs)** | | **2^nd^ degree**  **( 36 pairs)** | | **PO/FS**  **(146 pairs)** | | **Self-kinship**  **(254 individuals)** | |
| --- | --- | --- | --- | --- | --- | --- | --- | --- | --- | --- | --- |
|  |  | **RMSE** | **BIAS** | **RMSE** | **BIAS** | **RMSE** | **BIAS** | **RMSE** | **BIAS** | **RMSE** | **BIAS** |
| Bcftools | lcMLkin | 0.054 | 0.054 | 0.034 | 0.034 | 0.015* | 0.012* | 0.014* | -0.012* | -- | -- |
|  | GCTA | 0.005* | -0.004* | 0.033 | -0.033 | 0.065 | -0.065 | 0.124 | -0.124 | 0.048 | -0.045 |
|  | KING | 0.051 | 0.051 | 0.019* | -0.016* | 0.090 | -0.089 | 0.224 | -0.223 | -- | -- |
| BEAGLE | SEEKIN | 0.006 | -0.004 | 0.009* | -0.007* | 0.013* | -0.011* | 0.018* | -0.014* | 0.031 | -0.003* |
|  | GCTA | 0.005* | -0.003* | 0.020 | -0.019 | 0.036 | -0.035 | 0.065 | -0.064 | 0.019 | 0.015 |
|  | KING | 0.014 | -0.011 | 0.028 | -0.027 | 0.038 | -0.038 | 0.070 | -0.070 | -- | -- |
| BEAGLE+1KG3 | SEEKIN | 0.004* | -0.004 | 0.004* | -0.001* | 0.005* | -0.004* | 0.007* | -0.005* | 0.018 | -0.011* |
|  | GCTA | 0.004* | -0.003* | 0.009 | -0.008 | 0.014 | -0.013 | 0.022 | -0.022 | 0.015* | -0.014 |
|  | KING | 0.005 | 0.004 | 0.005 | -0.004 | 0.006 | -0.005 | 0.014 | -0.013 | -- | -- |

RMSE is the root mean squared error and BIAS is defined as the mean difference to the array-based estimates from PC-Relate for each type of relatedness. Negative values of BIAS suggest underestimation for results based on sparse sequencing data and vice versa.

^*^ Smallest magnitude of RMSE or BIAS in each call set and each type of relatedness
