# Supplementary material for: Estimation of kinship coefficient in structured and admixed populations using sparse sequencing data
Source: PLoS Genet. 2017 Sep 29;13(9):e1007021. doi: 10.1371/journal.pgen.1007021 (PMC5636172; doi:10.1371/journal.pgen.1007021)
Supplement: S3 Table — (DOCX) [file pgen.1007021.s004.docx]

**S3 Table. Performance of relationship classification based on homogeneous kinship estimators in ~0.75X sequencing data of 254 Chinese.**

| **Call set** | **Method** | **3^rd^ degree** | | **2^nd^ degree** | | **PO/FS** | |
| --- | --- | --- | --- | --- | --- | --- | --- |
|  |  | **Precision** | **Sensitivity** | **Precision** | **Sensitivity** | **Precision** | **Sensitivity** |
| Bcftools | lcMLkin | 0.001 | 0.773* | 0.844* | 1.000* | 1.000* | 1.000* |
|  | GCTA | 0.000 | 0.000 | 0.000 | 0.000 | -- | 0.000 |
|  | KING | 0.000 | 0.636 | -- | 0.000 | -- | 0.000 |
| BEAGLE | SEEKIN | 0.950* | 0.864* | 0.972* | 0.972* | 1.000* | 0.993* |
|  | GCTA | 0.348 | 0.364 | 0.438 | 0.583 | 1.000* | 0.815 |
|  | KING | 0.158 | 0.136 | 0.357 | 0.556 | 1.000* | 0.747 |
| BEAGLE+1KG3 | SEEKIN | 1.000* | 1.000* | 1.000* | 1.000* | 1.000* | 1.000* |
|  | GCTA | 0.950 | 0.864 | 1.000* | 0.972 | 1.000* | 1.000* |
|  | KING | 1.000* | 0.955 | 1.000* | 1.000 | 1.000* | 1.000* |

Precision is defined as the proportion of correct classification among all pairs of a relationship type inferred from the sequence-based kinship estimates. Sensitivity is defined as the proportion of correct classification among pairs of a relationship type inferred from the gold standard kinship estimates.

^*^ Highest values of precision or sensitivity in each call set and each relationship type.
