# Supplementary material for: Estimation of kinship coefficient in structured and admixed populations using sparse sequencing data
Source: PLoS Genet. 2017 Sep 29;13(9):e1007021. doi: 10.1371/journal.pgen.1007021 (PMC5636172; doi:10.1371/journal.pgen.1007021)
Supplement: S4 Table — (DOCX) [file pgen.1007021.s005.docx]

**S4 Table. Performance of heterogeneous kinship estimators in ~0.75X sequencing data of 762 Chinese and Malays.**

| **Call set** | **Method** | **Unrelated**  **(289,205 pairs)** | | **3^rd^ degree**  **(148 pairs)** | | **2^nd^ degree**  **(147 pairs)** | | **PO/FS**  **(437 pairs)** | | **Self-kinship**  **(762 individuals)** | |
| --- | --- | --- | --- | --- | --- | --- | --- | --- | --- | --- | --- |
|  |  | **RMSE** | **BIAS** | **RMSE** | **BIAS** | **RMSE** | **BIAS** | **RMSE** | **BIAS** | **RMSE** | **BIAS** |
| BEAGLE | SEEKIN | 0.005 | -0.002 | 0.008* | 0.004* | 0.010* | 0.006* | 0.021* | 0.015* | 0.045 | 0.022 |
|  | PC-Relate | 0.003* | 0.000* | 0.013 | -0.012 | 0.025 | -0.024 | 0.048 | -0.047 | 0.035 | 0.026 |
|  | REAP | 0.003* | -0.001 | 0.015 | -0.014 | 0.028 | -0.028 | 0.052 | -0.051 | 0.030* | 0.017* |
|  | RelateAdmix | 0.003* | 0.002 | 0.016 | -0.015 | 0.030 | -0.029 | 0.057 | -0.056 | -- | -- |
| BEAGLE+1KG3 | SEEKIN | 0.003 | -0.001 | 0.003* | 0.001* | 0.004* | 0.001* | 0.007* | 0.003* | 0.018 | 0.002* |
|  | PC-Relate | 0.002* | 0.000* | 0.005 | -0.004 | 0.010 | -0.009 | 0.020 | -0.020 | 0.017* | -0.014 |
|  | REAP | 0.002* | -0.001 | 0.008 | -0.008 | 0.014 | -0.014 | 0.024 | -0.023 | 0.018 | -0.017 |
|  | RelateAdmix | 0.002* | 0.001 | 0.005 | -0.005 | 0.009 | -0.009 | 0.016 | -0.016 | -- | -- |

RMSE is the root mean squared error and BIAS is defined as the mean difference to the array-based estimates from PC-Relate for each type of relatedness. Negative values of BIAS suggest underestimation for results based on sparse sequencing data and vice versa.

^*^ Smallest magnitude of RMSE or BIAS in each call set and each type of relatedness.
