# Supplementary material for: Estimation of kinship coefficient in structured and admixed populations using sparse sequencing data
Source: PLoS Genet. 2017 Sep 29;13(9):e1007021. doi: 10.1371/journal.pgen.1007021 (PMC5636172; doi:10.1371/journal.pgen.1007021)
Supplement: S5 Table — (DOCX) [file pgen.1007021.s006.docx]

**S5 Table. Performance of relationship classification based on heterogeneous kinship estimators in ~0.15X sequencing data of 762 Chinese and Malays.**

| **Call set** | **Method** | **3^rd^ degree** | | **2^nd^ degree** | | **PO/FS** | |
| --- | --- | --- | --- | --- | --- | --- | --- |
|  |  | **Precision** | **Sensitivity** | **Precision** | **Sensitivity** | **Precision** | **Sensitivity** |
| BEAGLE | SEEKIN | 0.934* | 0.858* | 0.916* | 0.959* | 1.000* | 0.986* |
|  | PC-Relate | 0.389 | 0.392 | 0.161 | 0.388 | 1.000* | 0.325 |
|  | REAP | 0.302 | 0.331 | 0.090 | 0.238 | 1.000* | 0.195 |
|  | RelateAdmix | 0.335 | 0.351 | 0.113 | 0.306 | 1.000* | 0.188 |
| BEAGLE+1KG3 | SEEKIN | 0.966* | 0.946* | 0.954* | 0.993* | 1.000* | 1.000* |
|  | PC-Relate | 0.867 | 0.791 | 0.866 | 0.878 | 1.000* | 0.954 |
|  | REAP | 0.758 | 0.635 | 0.796 | 0.796 | 1.000* | 0.931 |
|  | RelateAdmix | 0.836 | 0.723 | 0.900 | 0.857 | 1.000* | 0.968 |

Precision is defined as the proportion of correct classification among all pairs of a relationship type inferred from the sequence-based kinship estimates. Sensitivity is defined as the proportion of correct classification among pairs of a relationship type inferred from the gold standard kinship estimates.

^*^ Highest values of precision or sensitivity in each call set and each relationship type.
