# Supplementary material for: Estimation of kinship coefficient in structured and admixed populations using sparse sequencing data
Source: PLoS Genet. 2017 Sep 29;13(9):e1007021. doi: 10.1371/journal.pgen.1007021 (PMC5636172; doi:10.1371/journal.pgen.1007021)
Supplement: S6 Table — (DOCX) [file pgen.1007021.s007.docx]

**S6 Table. Performance of relationship classification based on heterogeneous kinship estimators in ~0.75X sequencing data of 762 Chinese and Malays.**

| **Call set** | **Method** | **3^rd^ degree** | | **2^nd^ degree** | | **PO/FS** | |
| --- | --- | --- | --- | --- | --- | --- | --- |
|  |  | **Precision** | **Sensitivity** | **Precision** | **Sensitivity** | **Precision** | **Sensitivity** |
| BEAGLE | SEEKIN | 0.964* | 0.905* | 0.935* | 0.980* | 1.000* | 1.000* |
|  | PC-Relate | 0.836 | 0.723 | 0.882 | 0.864 | 1.000* | 0.961 |
|  | REAP | 0.778 | 0.615 | 0.823 | 0.823 | 1.000* | 0.941 |
|  | RelateAdmix | 0.736 | 0.622 | 0.814 | 0.776 | 1.000* | 0.941 |
| BEAGLE+1KG3 | SEEKIN | 0.980* | 0.980* | 1.000* | 0.993* | 1.000* | 1.000* |
|  | PC-Relate | 0.959 | 0.953 | 1.000* | 0.966 | 1.000* | 0.998 |
|  | REAP | 0.948 | 0.858 | 0.986 | 0.952 | 1.000* | 0.995 |
|  | RelateAdmix | 0.965 | 0.919 | 1.000* | 0.966 | 1.000* | 1.000* |

Precision is defined as the proportion of correct classification among all pairs of a relationship type inferred from the sequence-based kinship estimates. Sensitivity is defined as the proportion of correct classification among pairs of a relationship type inferred from the gold standard kinship estimates.

^*^ Highest values of precision or sensitivity in each call set and each relationship type.
