# Supplementary figures and images for: Estimation of kinship coefficient in structured and admixed populations using sparse sequencing data
Source: PLoS Genet. 2017 Sep 29;13(9):e1007021. doi: 10.1371/journal.pgen.1007021 (PMC5636172; doi:10.1371/journal.pgen.1007021)

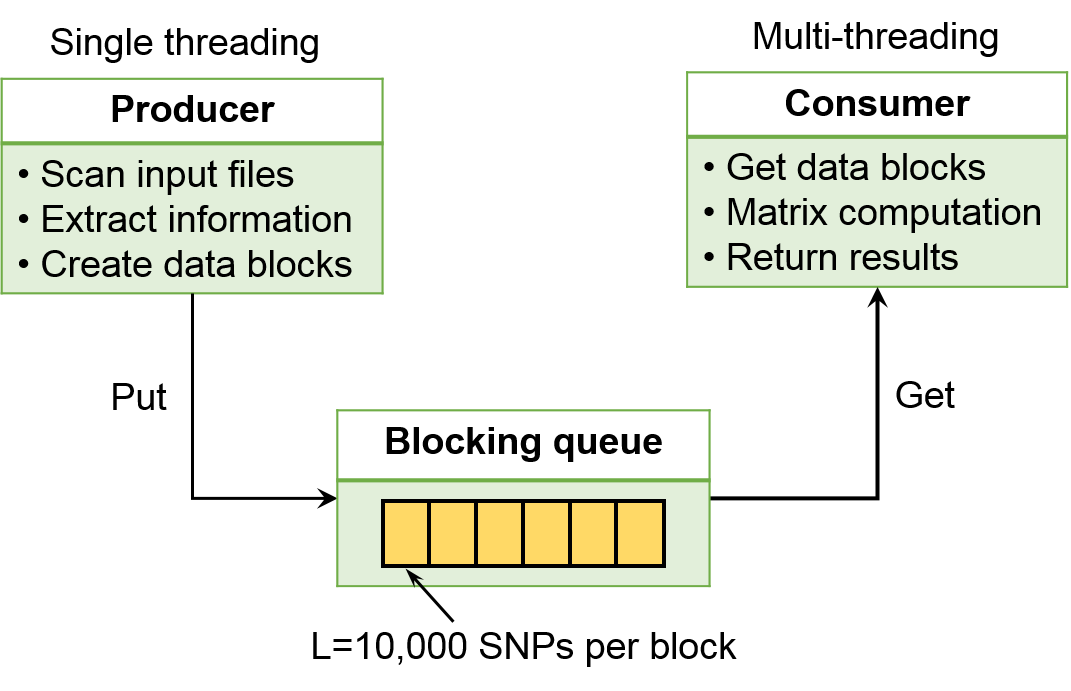

Supplement: S1 Fig — A single-threading “producer” job scans the input files, extracts required information for each SNP, and packs into a data block for every L SNPs. These data blocks are stored in the buffer, labeled as the blocking queue. Concurrently, a “consumer” job takes the data blocks one by one, performs multi-threading computation, and returns results. The results from different blocks are automatically combined after all blocks are analyzed. The “producer” and the “consumer” are synchronized through the blocking queue; the “producer” will become inactive if the blocking queue is full, and the “consumer” will become inactive if the blocking queue is empty. The best performance is achieved when production and consumption are balanced (i.e., the blocking queue is neither full nor empty). (TIF) [file pgen.1007021.s008.tif]

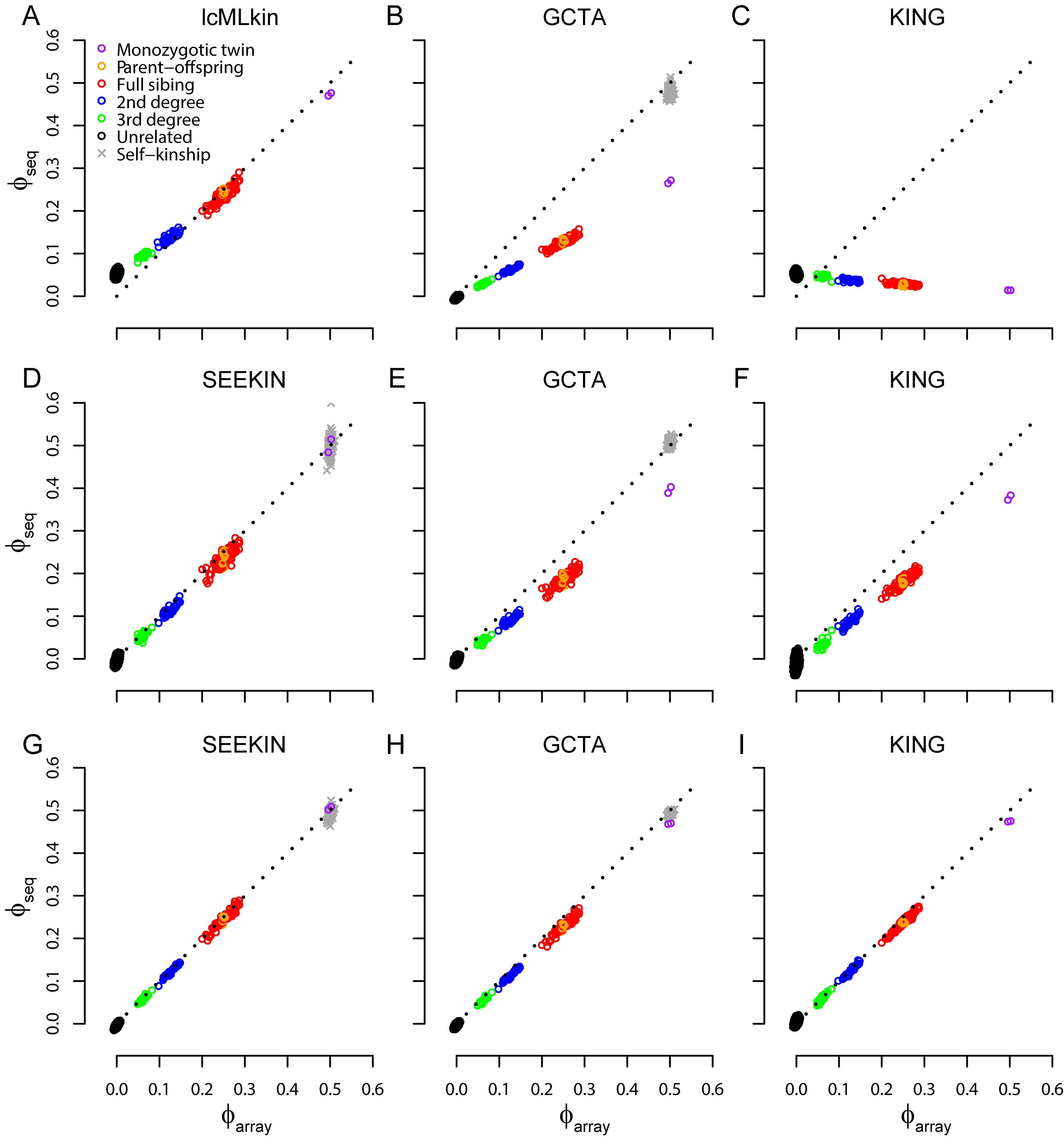

Supplement: S2 Fig — In each panel, we compared sequence-based estimates (ϕseq, y-axis) with the array-based estimates from PC-Relate (ϕarray, x-axis). Colored circles represent kinship coefficients between two individuals and different types of relatedness were determined in Fig 2. Grey crosses represent self-kinship coefficients. We evaluated lcMLkin (A), GCTA (B, E, H), KING (C, F, I), and SEEKIN (D, G) using the bcftools call set (A-C), the BEAGLE call set (D-F), and the BEAGLE+1KG3 call set (G-I). Note that KING does not estimate self-kinship coefficients. (TIF) [file pgen.1007021.s009.tif]

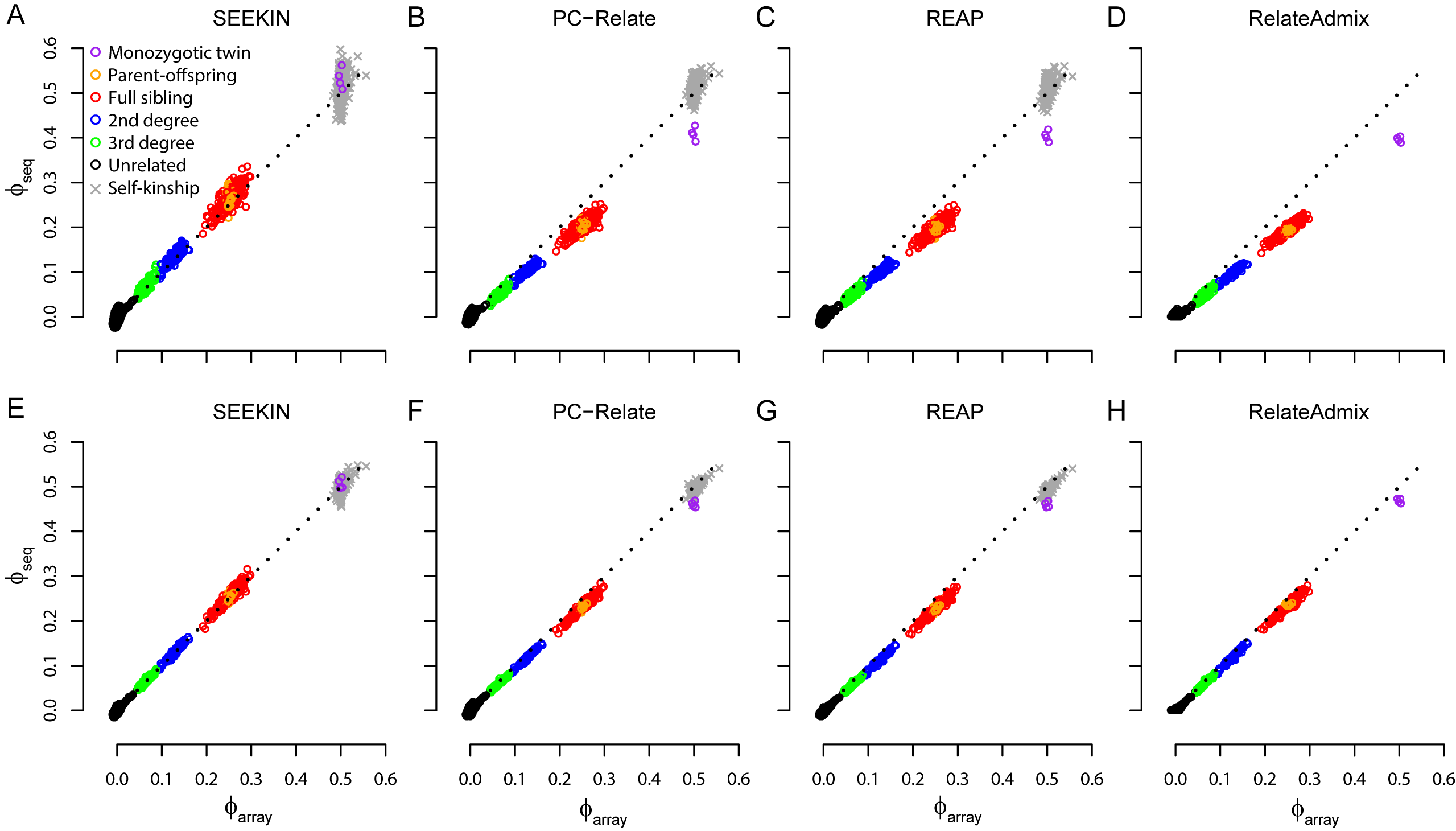

Supplement: S3 Fig — In each panel, we compared sequence-based estimates (ϕseq, y-axis) with the array-based estimates from PC-Relate (ϕarray, x-axis). Colored circles represent kinship coefficients between two individuals and different types of relatedness were determined in Fig 2. Grey crosses represent self-kinship coefficients. We evaluated SEEKIN (A, E), PC-Relate (B, F), REAP (C, G), and RelateAdmix (D, H) using the BEAGLE call set (A-D), and the BEAGLE+1KG3 call set (E-H). We only included SNPs overlapping with the SGVP dataset in the analyses, because we used the SGVP dataset as the reference panel to estimate individual-specific allele frequencies for SEEKIN, REAP and RelateAdmix. (TIF) [file pgen.1007021.s010.tif]

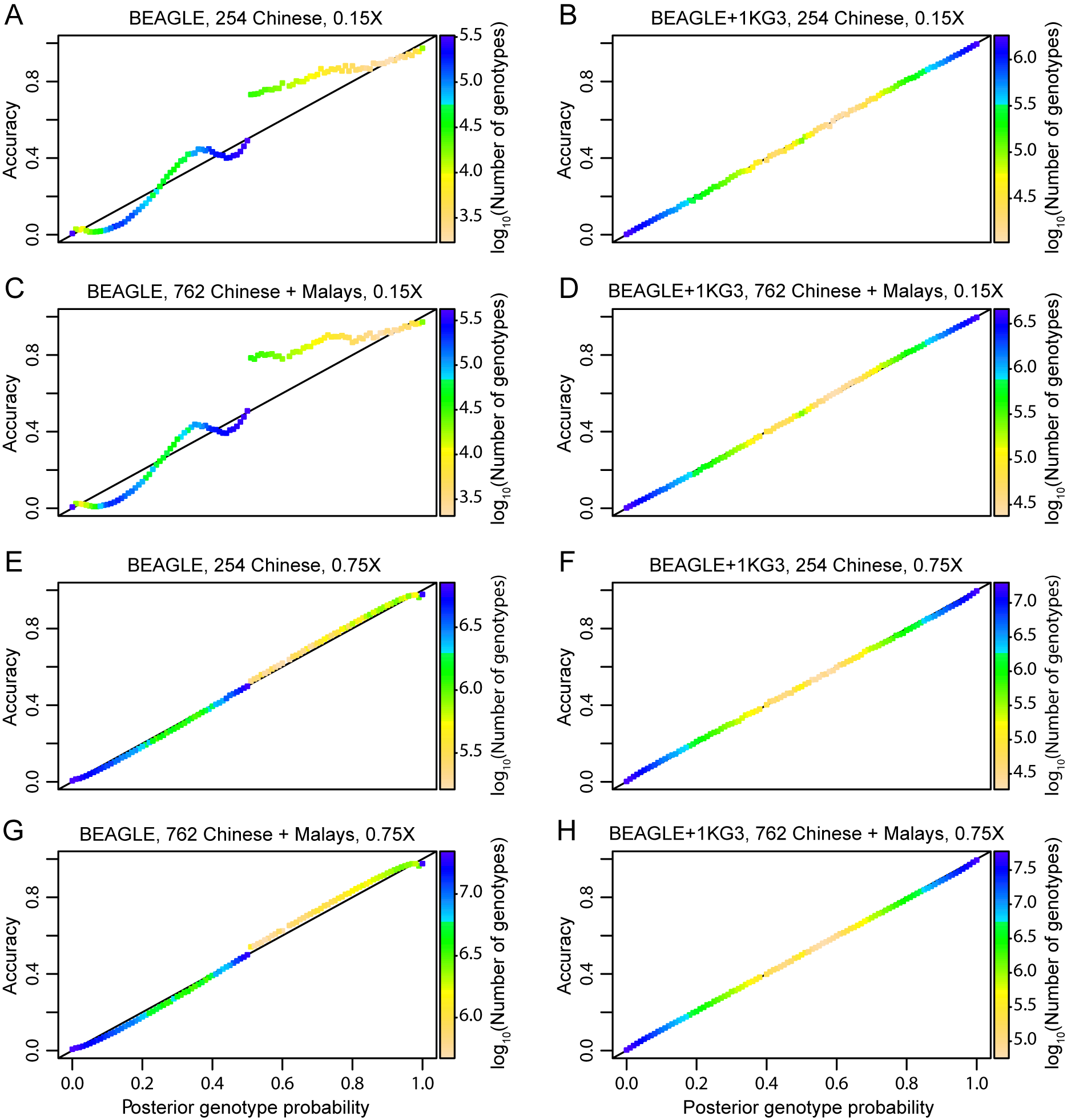

Supplement: S4 Fig — For each dataset, we binned the genotype probabilities into 100 bins spaced by 0.01 from 0 to 1 (x-axis). For each bin, we calculated the proportion of correct genotypes by comparing to the array genotypes (y-axis). The number of genotypes in each bin is color-coded according to the logarithmic scale in the color bar. When the genotype probabilities are well calibrated, we expect all data points on the diagonal. (A) BEAGLE call set for 254 Chinese at 0.15X. (B) BEAGLE+1KG3 call set for 254 Chinese at 0.15X. (C) BEAGLE call set for 762 Chinese and Malays at 0.15X. (D) BEAGLE+1KG3 call set for 762 Chinese and Malays at 0.15X. (E) BEAGLE call set for 254 Chinese at 0.75X. (F) BEAGLE+1KG3 call set for 254 Chinese at 0.75X. (G) BEAGLE call set for 762 Chinese and Malays at 0.75X. (H) BEAGLE+1KG3 call set for 762 Chinese and Malays at 0.75X. (TIF) [file pgen.1007021.s011.tif]

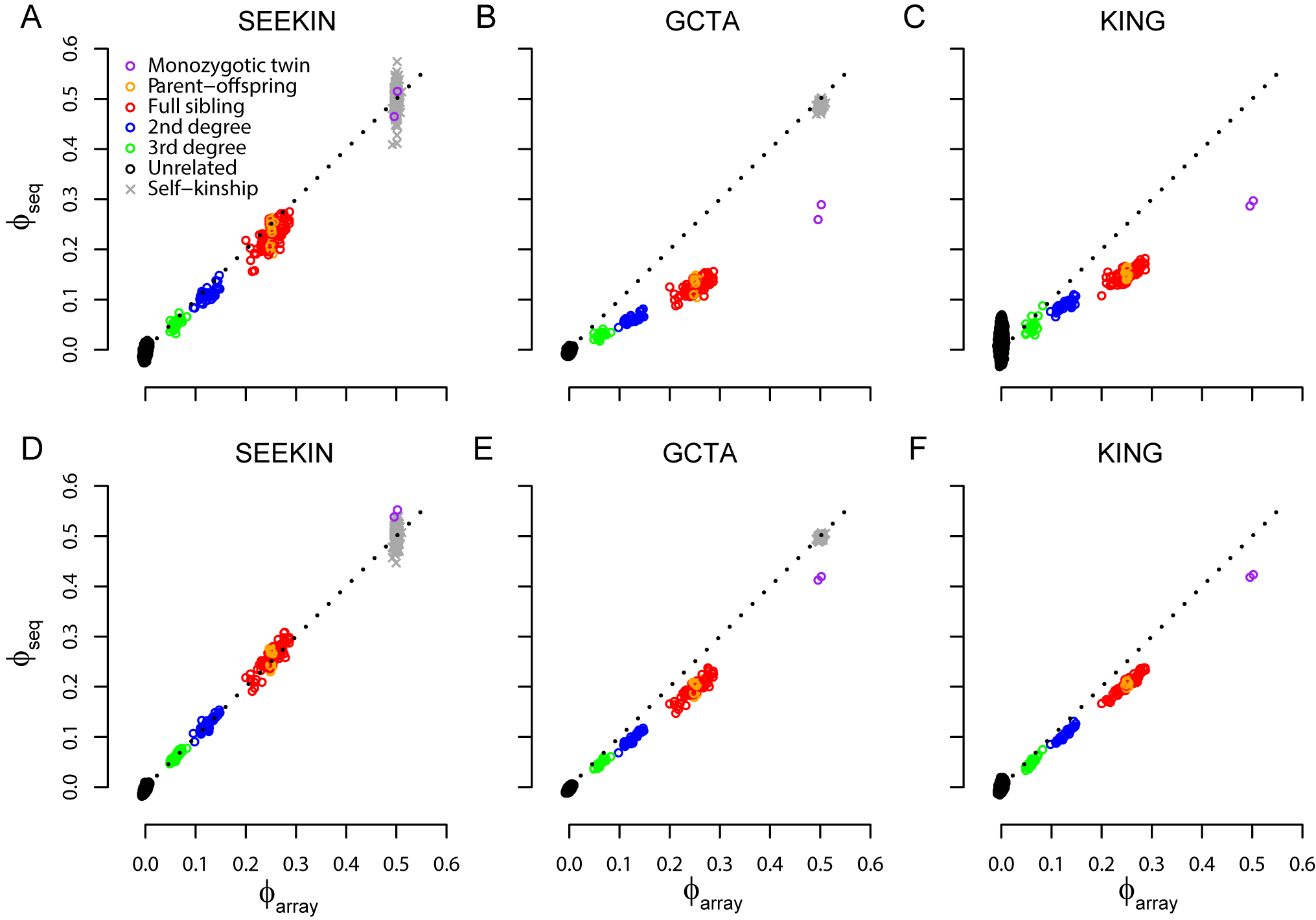

Supplement: S5 Fig — In each panel, we compared sequence-based estimates (ϕseq, y-axis) with the array-based estimates from PC-Relate (ϕarray, x-axis). Colored circles represent kinship coefficients between two individuals and different types of relatedness were determined in Fig 2. Grey crosses represent self-kinship coefficients. We evaluated SEEKIN (A, D), GCTA (B, E), and KING (C, F) using the BEAGLE call set (A-C), and the BEAGLE+1KG3 call set (D-F). Note that KING does not estimate self-kinship coefficients. (TIF) [file pgen.1007021.s012.tif]

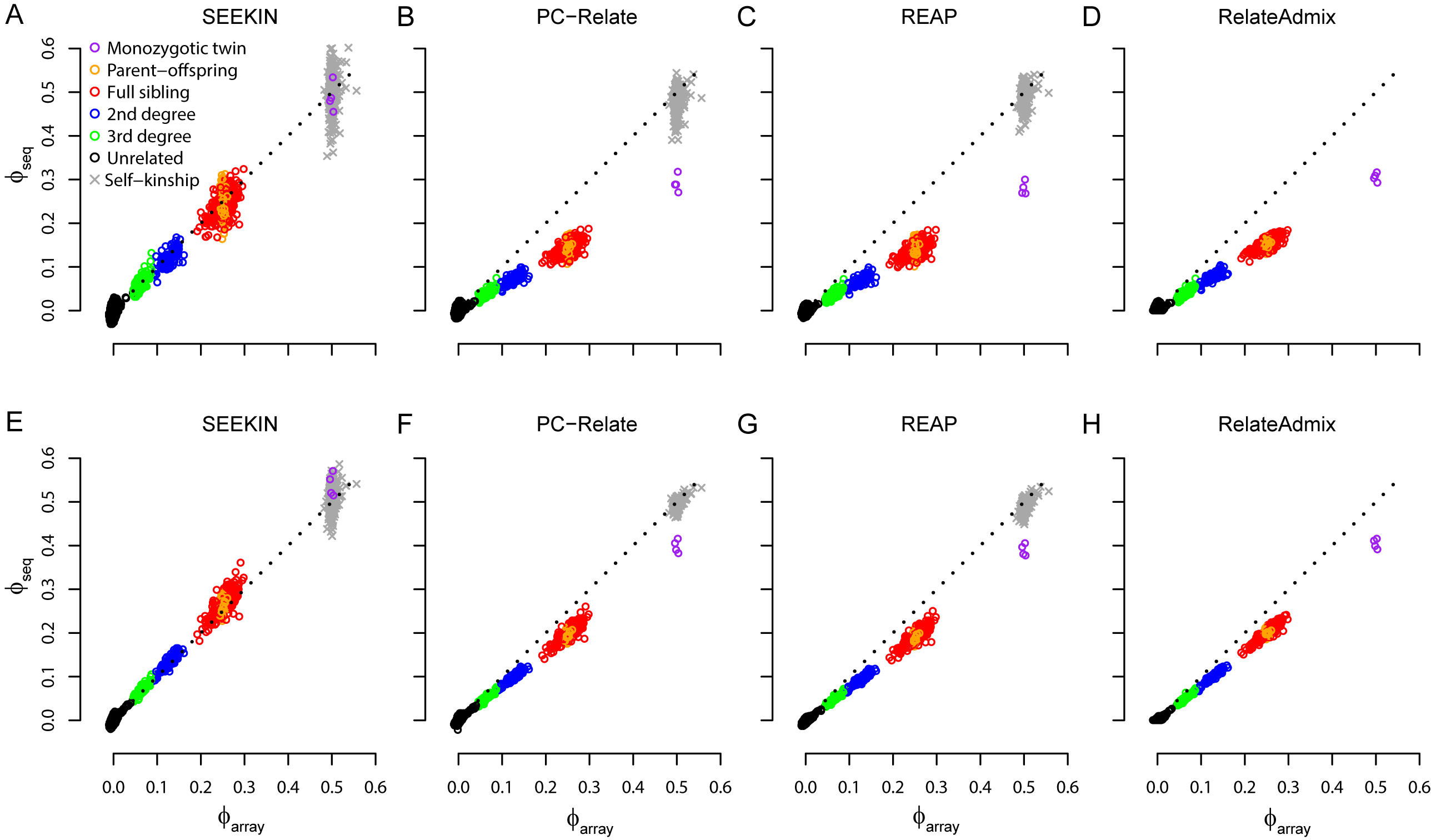

Supplement: S6 Fig — In each panel, we compared sequence-based estimates (ϕseq, y-axis) with the array-based estimates from PC-Relate (ϕarray, x-axis). Colored circles represent kinship coefficients between two individuals and different types of relatedness were determined in Fig 2. Grey crosses represent self-kinship coefficients. We evaluated SEEKIN (A, E), PC-Relate (B, F), REAP (C, G), and RelateAdmix (D, H) using the BEAGLE call set (A-D), and the BEAGLE+1KG3 call set (E-H). We only included SNPs overlapping with the SGVP dataset in the analyses, because we used the SGVP dataset as the reference panel to estimate individual-specific allele frequencies for SEEKIN, REAP and RelateAdmix. (TIF) [file pgen.1007021.s013.tif]
